# GAGA factor, a positive regulator of global gene expression, modulates transcriptional pausing and organization of upstream nucleosomes

**DOI:** 10.1186/s13072-016-0082-4

**Published:** 2016-07-27

**Authors:** Shih-Ying Tsai, Yuh-Long Chang, Krishna B. S. Swamy, Ruei-Lin Chiang, Der-Hwa Huang

**Affiliations:** 1 Institute of Molecular Biology, Academia Sinica, Nankang, Taipei Taiwan, ROC; 2 Molecular Cell Biology, Taiwan International Graduate Program, Institute of Molecular Biology, Academia Sinica, Graduate Institute of Life Sciences, National Defense Medical Center, Taipei, Taiwan, ROC

**Keywords:** GAGA factor, RNA polymerase II, Transcription pausing, Nucleosome

## Abstract

**Background:**

Genome-wide studies in higher eukaryotes have revealed the presence of paused RNA polymerase II (RNA-Pol) at about 30–50 bp downstream of the transcription start site of genes involved in developmental control, cell proliferation and intercellular signaling. Promoter-proximal pausing is believed to represent a critical step in transcriptional regulation. GAGA sequence motifs have frequently been found in the upstream region of paused genes in *Drosophila*, implicating a prevalent binding factor, GAF, in transcriptional pausing.

**Results:**

Using newly isolated mutants that retain only ~3 % normal GAF level, we analyzed its impacts on transcriptional regulation in whole animals. We first examined the abundance of three major isoforms of RNA-Pol on *Hsp70* during heat shock. By cytogenetic analyses on polytene chromosomes and chromatin immunoprecipitation (ChIP), we show that paused RNA-Pol of *Hsp70* is substantially reduced in mutants. Conversely, a global increase in paused RNA-Pol is observed when GAF is over-expressed. Coupled analyses of transcriptome and GAF genomic distribution show that 269 genes enriched for upstream GAF binding are down-regulated in mutants. Interestingly, ~15 % of them encode transcriptional factors, which might control ~2000 additional genes down-regulated in mutants. Further examination of RNA-Pol distribution in GAF targets reveals that a positive correlation exists between promoter-proximal RNA-Pol density and GAF occupancy in WT, but not in mutants. Comparison of nucleosome profiles indicates that nucleosome occupancy is preferentially attenuated by GAF in the upstream region that strongly favors nucleosome assembly. Using a dominant eye phenotype caused by GAF over-expression, we detect significant genetic interactions between GAF and the nucleosome remodeler NURF, the pausing factor NELF, and BAB1 whose binding sites are enriched specifically in genes displaying GAF-dependent pausing.

**Conclusion:**

Our results provide direct evidence to support a critical role of GAF in global gene expression, transcriptional pausing and upstream nucleosome organization of a group of genes. By cooperating with factors acting at different levels, GAF orchestrates a series of events from local nucleosome displacement to paused transcription. The use of whole animals containing broad tissue types attests the physiological relevance of this regulatory network.

**Electronic supplementary material:**

The online version of this article (doi:10.1186/s13072-016-0082-4) contains supplementary material, which is available to authorized users.

## Background

Eukaryotic transcription requires coordinated activities from distinct ensembles acting directly on transcriptional processes or chromatin architecture. Previous studies have shown that the transcriptional machinery undergoes stepwise catalytic transitions from initial assembly to termination upon the integration of regulatory information from general and gene-specific factors, while the overall organization and constituents of chromatin are dynamically modified to facilitate loading and progression of the transcriptional machinery [1]. Despite a wealth of knowledge on the interplay of these activities, new features continue to emerge and await further studies.

Analyses in cell-free systems have established that the supramolecular assembly of transcriptional complexes over the transcription start site (TSS) represents a critical rate-limiting step [2]. However, in vivo studies of *DrosophilaHeat shock 70* (*Hsp70*) and human c-Myc and c-Fos genes have revealed that RNA polymerase II (RNA-Pol) may stall for lengthy periods at about 30–50 bp downstream of the TSS, implicating a novel rate-limiting step prior to productive transcription [3–6]. Subsequently, this promoter-proximal pausing has widely been found in genes involved in developmental regulation, cell proliferation and intercellular signaling from fly to human [7–11]. It has been proposed that pausing is responsible for rapid and synchronous transcriptional induction [12, 13]. One distinguishing feature of the paused RNA-Pol is the presence of hyper-phosphorylated serine 5 residues (Ser-5p) in heptad repeats of Rpb1’s C-terminal domain (CTD). In contrast, RNA-Pol engaged in pre-initiation is hypo-phosphorylated (Hypo-p), while the productively elongating form gains additional phosphorylation on serine 2 residues (Ser-2p) [14, 15]. Both biochemical and genomic studies have further shown that paused RNA-Pol is stabilized by its association with NELF (negative elongation factor) and DSIF (DRB sensitivity-inducing factor) and that phosphorylation of these factors by a cyclin-dependent kinase, P-TEFb (positive transcription elongation factor b), triggers the transition of RNA-Pol from paused to elongation states [16–21].

Several features have also been noted for paused genes. For example, the TSS of these genes is clustered in narrow regions [7], which are frequently associated with a particular set of core promoter elements [22–24]. Certain nucleotide compositions are enriched in sequences immediately upstream or downstream of TSS [7, 23]. Furthermore, consistent with the view that nucleosomes act as a transcription barrier, paused RNA-Pols have been shown to contact the first nucleosome of the highly ordered downstream nucleosomal array [25]. Paradoxically, a closer examination of genes with different degrees of pausing has revealed that highly paused genes tend to possess lower levels and less organized downstream nucleosomes than those with weaker or no pausing [23]. Recent studies have shown that these differences may reflect the occupancy of specific upstream binding factors, suggesting the involvement of distinct mechanisms for transcriptional pausing [26].

A large fraction of paused genes, including *Hsp70,* contain GAGA sequence motifs in the upstream region in *Drosophila*. Two sequence-specific DNA binding proteins, GAF and Psq factor, are known to bind this motif [27, 28]. Genome-wide studies have revealed a correlation between GAF occupancy and paused genes [22, 29, 30]. GAF consists of two structurally and functionally similar isoforms containing an N-terminal POZ/BTB protein interaction domain, a single zinc finger capable of sequence-specific binding and a glutamine-rich C-terminal domain [31–33]. Several, sometimes incompatible, in vitro and in vivo functions have been assigned to GAF, including anti-silencing, transcriptional activation, heterochromatin-mediated silencing and Hox gene activation or repression [28, 34–37]. Moreover, its association with NURF (nucleosome remodeling factor) and dFACT (facilitates chromatin transcription) suggests a functional link to chromatin organization [38, 39]. Recently, it has been shown that GAF knockdown in a cultured cell line results in reduced pausing and increased nucleosomes in both upstream and downstream regions [40]. Whether these effects are exerted in other cells and, more importantly, relevant in the living organism remain unclear.

Here we used newly isolated *Gaf* mutants to examine their effects on gene regulation in whole animals. We started with transcriptional analyses of *Hsp70* and extended to genome-wide studies. We found that GAF, as an activator required for global gene expression, modulates the level of paused RNA-Pol and the nucleosome pattern specifically in the region immediately upstream of the TSS by cooperating with various sequence-independent and sequence-specific factors.

## Results

### Selective reduction of RNA-Pol Ser-5p on *Hsp70* in *Gaf* mutants

**Fig. 1:**
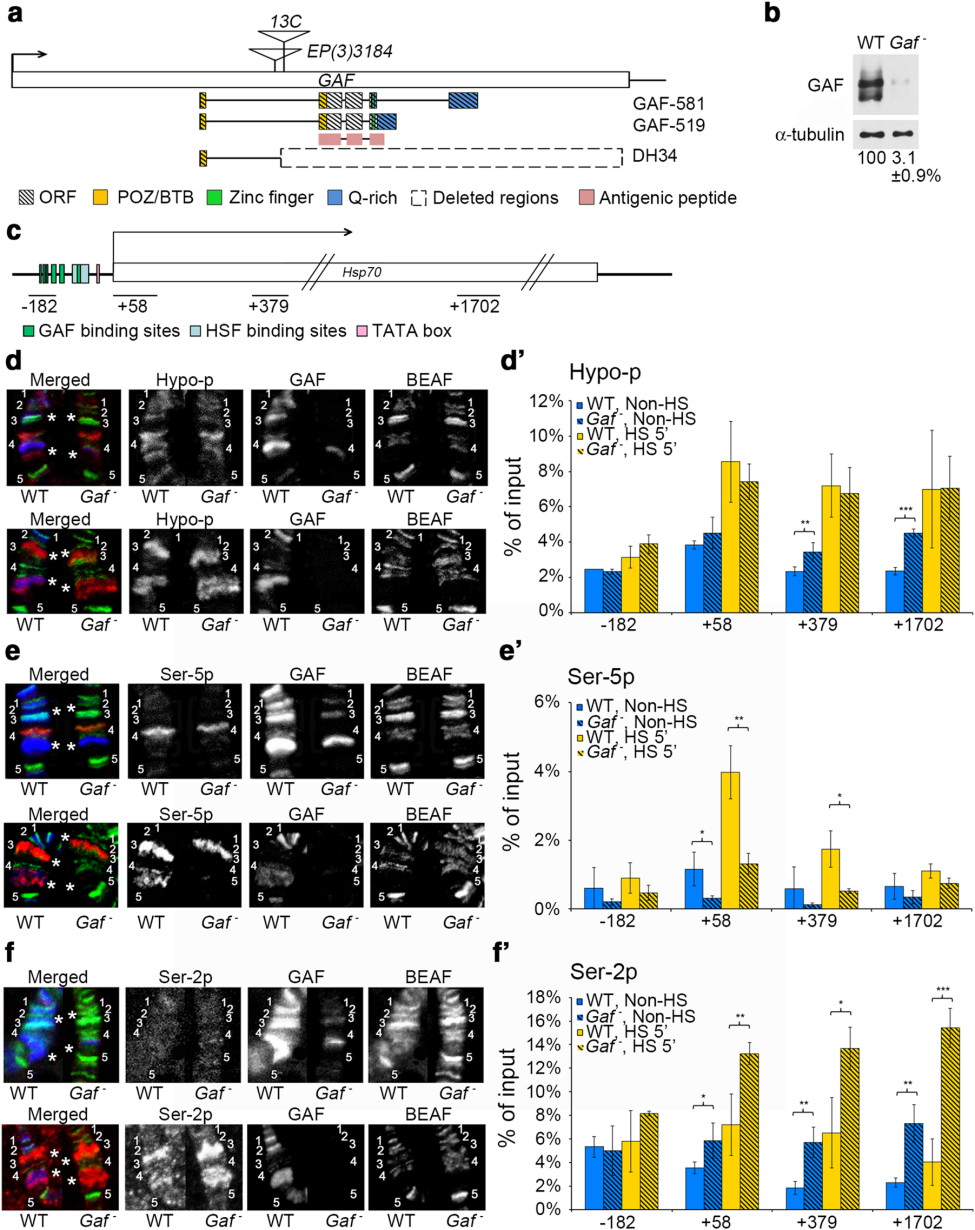
GAF modulates different phosphoisoforms of RNA-Pol at the *Hsp70* locus. **a** Molecular map of the *Gaf* locus. Two isoforms of GAF, GAF-519 and GAF-581, share the same BTB/POZ (*yellow box*) and Zn-finger domains (*green box*), but differ in the Gln-rich domain (*blue box*). The GAF antibody is raised against the shared region (*pink box*). The P-element insertion lines, *Gaf* ^*13C*^ and *EP(3)3184*, are indicated b*y triangles*. The deletion in *Gaf* ^*DH34*^, shown by the *dotted box*, extends to 218 bp upstream of 3′end of the gene. **b** Quantification of GAF. Extracts from salivary glands of third-instar larvae were immunoblotted with GAF and α-tubulin antibodies. The relative abundance of GAF from duplicate experiments is shown. **c** Gene map of *Hsp70.* A simplified map is shown with approximate locations of the GAF and HSF binding sites, TATA box, and four sets of primers used for qPCR. The relative position to the TSS for the midpoint of each PCR fragment is indicated. **d**–**f**. Cytogenetic studies of RNA-Pol at *Hsp70* loci. Polytene chromosomes from WT and mutants before (**d**–**f**, *upper panels*) or after a 30-min heat shock (**d**–**f**, *lower panels*) were simultaneously stained with antibodies against RNA-Pol (Hypo-p, Ser-5p or Ser-2p *in red*), GAF (*in blue*) and BEAF-32 (*in green*). The *color* is removed for panels showing individual staining for clarity. The region from 87A to 87C is marked by five sections (1–5) based on BEAF-32 staining. Two *Hsp70* clusters are located in sections 2–3 and 4–5 and are indicated by *. **d**’–**f**’. Quantitative measurement of RNA-Pol on *Hsp70.* Chromatin samples prepared from imaginal tissues of WT or *Gaf* mutant larvae before (non-HS) or after treatment at 37 °C for 5 min (HS) were subjected to immunoprecipitation with RNA-Pol antibodies against Hypo-p (**d**’), Ser-5p (**e**’), or Ser-2p (**f**’), followed by qPCR using four primer sets shown in **c**. The amounts of qPCR products from each set of measurements are expressed as a percentage of input. The average of three ChIP experiments is shown. Statistically significant differences are indicated (*t* test, *p < 0.05; **p < 0.01; ***p < 0.001)

To investigate the role of GAF in transcriptional regulation, we first examined the phosphorylation status of two key serine residues (i.e., Ser-2 and Ser-5) in CTD of RNA-Pol in wild type (WT) and *Gaf* mutant larvae. The GAF-coding gene was previously designated as *trithorax*-*like* (*Trl*) residing in the left arm of the third chromosome, based on the observation that some mutant alleles could cause homeotic transformations like *trithorax* [36]. However, this assignment was not supported by subsequent reports [41, 42]. In fact, we found that the homeotic effect is lost when the right arm of the third chromosome of original alleles is replaced by the genetically marked WT chromosome (unpublished data). To avoid potential confusion, we generated a new set of deletion mutants from a homozygous viable P insertion line, *EP3184* (Fig. 1a), and referred them as *Gaf* alleles. In addition, we removed the putative second-site mutation from *Trl*^*13C*^ by recombination and designated it as *Gaf*^*13C#3*^. Homozygous *Gaf* deletion mutations are lethal during early larval development, while *Trl*^*13C*^ and *Gaf*^*13C#3*^ can develop into sterile adults. However, the combination of *Gaf*^*DH34*^—an allele in which sequences from the *EP3184* insertion site to the 3′ non-coding region of *Gaf* have been deleted (Fig. 1a)—and *Gaf*^*13C#3*^ allows animals to survive until early pupal stage. Using a purified antibody raised against the common region of GAF isoforms (Fig. 1a, pink region), we detected ~3 % WT level of proteins in *Gaf*^*DH34*^*/Gaf*^*13C#3*^ trans-heterozygotes during the late third instar (Fig. 1b). To facilitate our analyses, we used these trans-heterozygotes for subsequent studies unless otherwise specified.

Previous studies have shown that RNA-Pol undergoes both qualitative and quantitative changes during heat induction of *Hsp70* genes [43]. RNA-Pol is initially paused at the region ~50 bp downstream of the TSS (+50) primarily with Ser-5p, but is rapidly converted into the isoform actively engaged in transcriptional elongation with additional phosphorylation on Ser-2. To understand how this process is regulated by the binding of GAF to the upstream GAGA repeats, we analyzed the relationship between GAF and the phosphorylation state of RNA-Pol following heat induction of *Hsp70*. *Drosophila* contains six *Hsp70* genes located in two cytogenetically discernible clusters at map positions 87A and 87C on polytene chromosomes. We used simultaneous staining of GAF and BEAF-32—a boundary element-binding protein [44, 45]—as controls for staining quality. In addition, the major bands of BEAF-32 staining were used to mark this region with five sections (1–5, Fig. 1d–f) for further comparison. The 87A region contains two *Hsp70* genes located between sections 2 and 3, and the 87C region contains four *Hsp70* genes between sections 4 and 5 (marked by stars). Before induction, the signals of RNA-Pol isoforms including Hypo-p, Ser-5p and Ser-2p were either very weak or undetectable on these locations of both WT and mutant chromosomes (Fig. 1d–f, upper panel). Following 30-min heat induction, strong signals of all three isoforms appeared at both the 87A and 87C clusters. No significant difference was observed for the Hypo-p signal between WT and mutant chromosomes (Fig. 1d, lower panel), but a substantial reduction in the Ser-5p signal was evident on mutant chromosomes (Fig. 1e, lower panel). In addition, the Ser-2p signal appeared to increase significantly on mutant chromosomes (Fig. 1f, lower panel).

To confirm these cytogenetic results, we used chromatin immunoprecipitation (ChIP) to quantitatively analyze the phosphorylation profile of RNA-Pol on *Hsp70*. Cross-linked chromatin samples prepared from third-instar larvae containing imaginal disks, nerve cords and salivary glands were subjected to ChIP and quantitative PCR (qPCR) to measure the relative abundance of these isoforms in three intragenic regions centered at +58, +379 and +1702 as well as an upstream region centered at −182 for comparison (Fig. 1c). Prior to heat shock, both Hypo-p and Ser-5p signals were somewhat higher around +58 in WT samples, in agreement with the observation of paused RNA-Pol at the promoter-proximal region [46]. In mutant samples, although the level of Hypo-p did not show significant change in this region, relatively lower level of Ser-5p was seen (Fig. 1e’), suggesting the reduction of paused RNA-Pol in the absence of GAF. Interestingly, the Ser-2p signal showed substantial increases in the distal part of *Hsp70*, indicating that productive transcription occurs concomitantly in mutants (Fig. 1f’). We noted that there were also moderate increases in Hypo-p in downstream regions (Fig. 1d’), which presumably reflect incomplete phosphorylation of RNA-Pol during elongation [47].

Upon a brief heat shock (i.e., 5 min), WT samples showed substantial increases in signal intensity for all three RNA-Pol isoforms at the +58 region and further downstream, as expected for fully active transcription. The increase in the Ser-5p signal was most pronounced at the promoter-proximal region, indicating the accumulation of considerable amounts of paused RNA-Pol. Clearly, the transition of RNA-Pol from paused to elongation states remains rate limiting during transcriptional activation. Similar to the uninduced condition, the Ser-5p signal showed a drastic reduction from WT to mutant samples. Again, the Ser-2p signal in mutant samples showed concomitant increases in the coding region, particularly at the 3′ end. Despite these changes, no significant differences were found between WT and mutant samples for total RNA-Pol, as measured on polytene chromosomes using an antibody recognizing the Rpb3 subunit of RNA-Pol (Additional file 1: Fig. S1A, B). These results confirm our cytogenetic observations and together indicate that paused RNA-Pol becomes more frequently released to engage in elongation in the absence of GAF. Thus, the transition between promoter-proximal pausing and productive elongation of RNA-Pol appears to be modulated by GAF.

### Global regulation of RNA-Pol phosphorylation

**Fig. 2:**
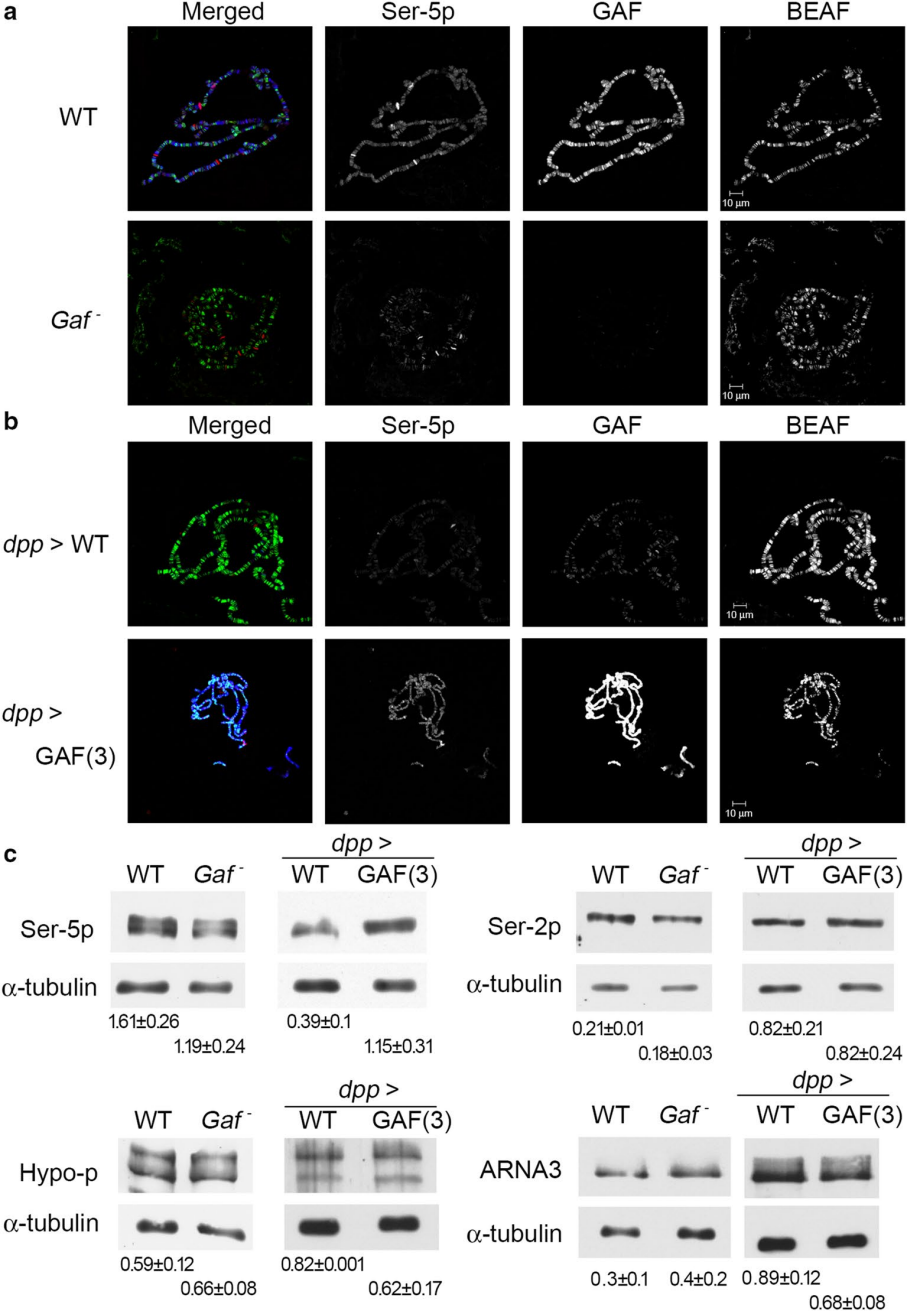
Global effects of GAF on the level of RNA-Pol Ser-5p. **a** Reduction of RNA-Pol Ser-5p in *Gaf* mutants. Polytene chromosomes from WT and *Gaf* mutants were co-stained and processed as described in Fig. 1. **b** Increase in RNA-Pol Ser-5p by GAF over-expression. Polytene chromosome from *dpp* > WT or *dpp* > GAF(3) samples were co-stained as described in Fig. 1. **c** Detection of differential effects of GAF on RNA-Pol phosphoisoforms. Salivary gland extracts from WT, *Gaf* mutants and *dpp* > GAF(3) lines were immunoblotted with antibodies against three RNA-Pol phosphoisoforms, total RNA-Pol (ARNA3) and α-tubulin. The abundance of each RNA-Pol isoform was adjusted with that of α-tubulin. The relative abundance of each RNA-Pol isoform from triplicate experiments is shown

We next asked whether the global level of various forms of phosphorylated RNA-Pol could be affected by either *Gaf* mutation or GAF over-expression. Again, we used GAF and BEAF-32 staining as internal controls for polytene chromosome staining. Compared to WT chromosomes, the overall intensity of Hypo-p and Ser-2p staining did not show any significant change on mutant chromosomes (Additional file 1: Fig. S2A, B). In contrast, the overall intensity of Ser-5p was reduced in mutant chromosomes (Fig. 2a). In the complementary study, we drove over-expression of a GAF isoform, GAF-519, in salivary glands by introducing *dpp*-Gal4 to a transgenic line containing UAS-GAF on third chromosome (henceforth called *dpp* > GAF(3)). The resultant chromosomes were intensely labeled with GAF signals and were thinner and more difficult to spread than WT ones. In proportion to the reduced chromosomal size, the overall BEAF-32 signals also appeared to be relatively weaker. Importantly, there was a substantial increase in Ser-5p signal (Fig. 2b). However, no obvious changes were observed for Hypo-p, Ser-2p and total RNA-Pol signals (Additional file 1: Figs. S1C and S2C, D).

The relative amounts of different RNA-Pol isoforms were further measured by immunoblotting, using extracts prepared from salivary glands of these larvae. While there was a modest reduction of Ser5-p signal in mutant extracts, a substantial increase in Ser-5p (~threefold) signal was observed for *dpp* > GAF(3) extracts. In contrast, both Hypo-p and Ser2-p did not show significant differences in *dpp* > GAF(3) extracts (Fig. 2c). To further determine whether the changes of Ser5-p might reflect the amount of total RNA-Pol under different genetic backgrounds, we also examined these extracts with ARNA-3 antibody that recognizes an epitope distant from the CTD [48]. In contrast to Ser5-p, total RNA-Pol was slightly increased in mutant extracts, but slightly reduced in *dpp* > GAF(3) extracts (Fig. 2c, and Additional file 1: Fig. S1D), further revealing the selective effects on Ser-5p. These results strongly support a global role of GAF on regulation of Ser-5p.

### GAF controls global gene expression

**Fig. 3:**
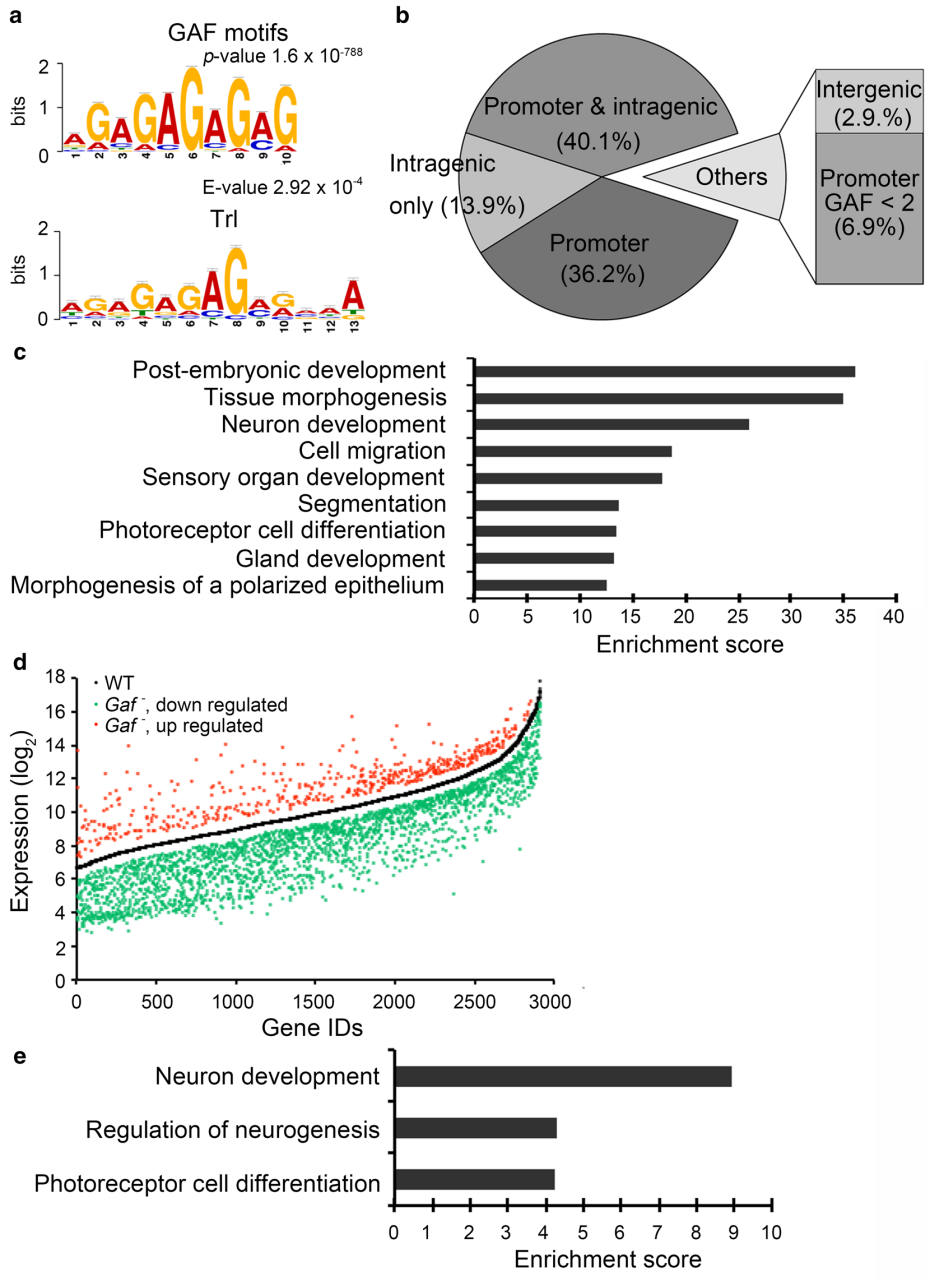
Genomic analyses of GAF target genes and *Gaf* mutation on gene expression. **a** GAF binding motif. The sequences of GAF peaks were analyzed by MEME. The most significant motif is shown with the statistical value (*p* value = 1.6 × 10^−788^). The statistical comparison with previously identified Trl motif is also shown (*E* value = 2.92 × 10^−4^). **b** Summary of GAF distribution. GAF binding sites identified by ChIP-sequencing and evaluated by statistical analyses are summarized in a pie chart. GAF peaks with more than twofold enrichment over the input are grouped into four categories: promoter only (from −500 to TSS), both promoter and intragenic region (UTR, intron and exon), intragenic region only, and intergenic region (beyond −500 or 3’ end). A small percentage of GAF peaks with less than twofold enrichment in the promoter region are also indicated. **c** Biological functions enriched in GAF targets. Putative GAF-target genes were subjected to DAVID for functional annotation clustering analysis. The GO term and the enrichment score of clusters above 10 (FDR < 0.0001) are shown. **d** The global effect of *Gaf* mutation on gene expression. Triplicate RNA samples from WT and *Gaf* mutants were subjected to microarray analysis. A scatter plot summarizes statistically significant changes in gene expression (*p* < 0.05 and >1.5-fold change). Each gene is assigned an ID number according to the ascending order of its expression level as shown in the log_2_ scale in the *y*-axis. The central curve represents the WT expression level of genes that show significant changes in mutants. Approximately 80 or 20 % of genes show reduced (*green*) or increased expression (*red*) in mutants, respectively. **e** GO analysis of GAF targets with affected expression in mutants. GAF targets with significant changes in expression were subjected to analysis for functional clustering. The GO term and the enrichment score of clusters above 2 (FDR < 0.05) are shown

To further elucidate the role of GAF in global transcriptional regulation, we next examined the genome-wide distribution of GAF in larval tissues. Chromatin prepared from WT imaginal tissues of third-instar larvae was subjected to ChIP with an affinity-purified GAF antibody, followed by deep sequencing (ChIP-seq) to get more than thirty million reads (~32 × 10^6^ for GAF, ~34 × 10^6^ for input), which were used for further analyses. Using the PICS program (Probabilistic inference for ChIP-Seq) [49], we identified 3716 GAF binding peaks (FDR < 0.05). Subsequent analysis by the MEME (Multiple EM for Motif Elicitation) program revealed a reiterated GA motif with the highest score (*p* = 1.6 × 10^−788^) [50]. Although this motif is similar to the known consensus Trl binding sequences (E = 2.92 × 10^−4^ by TOMTOM analysis) (Fig. 3a), it appears to conform much better to the (GA)n signature. However, it is worth noting that about 80 % of such sequences in the genome lack significant amounts of GAF binding (data not shown), indicating that not all sequences are equally accessible.

**Fig. 4:**
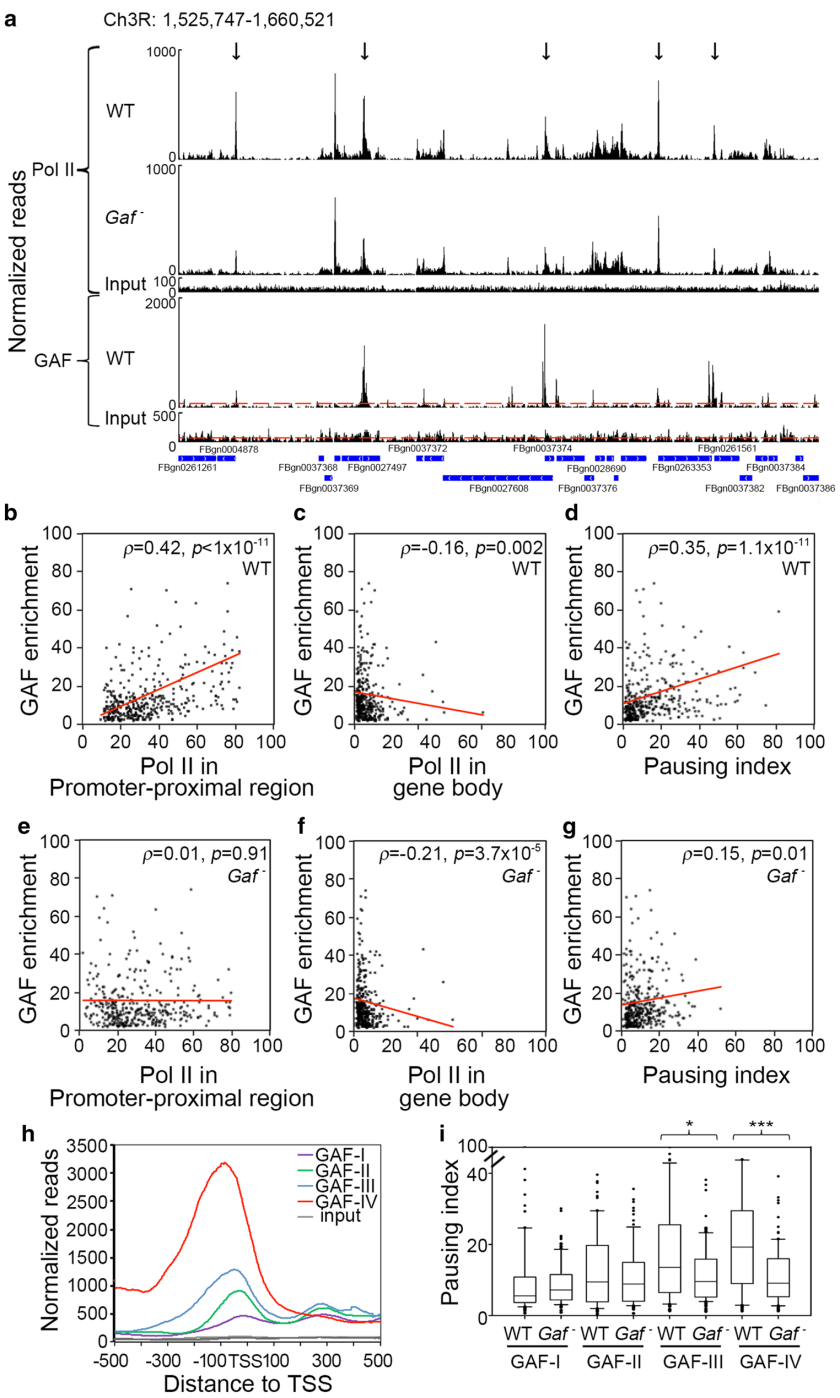
Reduction of paused RNA-Pol in *Gaf* mutants. **a** Distribution of RNA-Pol and GAF in the 83B-C region. RNA-Pol and GAF reads at each nucleotide were normalized with the total reads of each sample. The cumulative reads of RNA-Pol and GAF in the section from 1,525,747 to 1,660,521 of the third chromosome are shown with the gene map below. Genes with significant reduction of promoter-proximal RNA-Pol in mutants are indicated by *arrows*. Note that input reads are significantly higher than background reads in ChIP samples, potentially reducing the number of enriched peaks. The average read for the input sample is 70. *Red dash lines* marking this value in GAF ChIP and input are shown in GAF * panel*. **b**–**g** Correlation between RNA-Pol and GAF. RNA-Pol density in the promoter-proximal region (**b**, **e**; from −100 to +150) and gene body (**c**, **f**; from +500 to the end) in WT (**b**, **c**) or mutants (**e**, **f**) is separately plotted against the WT level of GAF occupancy according to the enrichment score of each promoter. PIs calculated from the ratio of RNA-Pol densities in the promoter-proximal region over the gene body are similarly plotted for WT (**d**) and mutants (**g**). Three hundred and sixty-five genes were included in this analysis. Statistical values for Spearman’s rank correlation coefficient (ρ) and *p* value (*p*) are shown. **h** GAF distribution in different ranks of targets. Three hundred and sixty-five GAF targets are divided into four ranks based on the overall GAF enrichment score from −500 to TSS. Ranks I to IV range from lowest to highest GAF scores. GAF distribution from −500 to +500 is shown for each rank. The normalized reads of both GAF peaks and input are shown in *y*-axis. **i** Dosage-dependent relationship between RNA-Pol pausing and GAF occupancy. The PIs of each rank of genes in WT and mutants are represented by *box plots*. The *whiskers* represent 5 and 95 % of each rank. The statistical significance of differences calculated by Mann–Whitney *U* test is indicated for ranks III (*p* = 0.03, *) and IV (*p* = 6.04 × 10^−5^, ***)

These GAF binding peaks could be assigned to 1891 annotated genes, which are referred to as putative GAF-target genes. Among them, 1442 genes (~76 %) contain GAF peaks with values at least twofold higher than the corresponding regions in the input control (Fig. 3b). Importantly, they all contain GAF peaks within 500 bp upstream of the TSS. About half of them have additional peaks located within the gene body including exons and introns. For genes with less GAF occupancy (<twofold enrichment), their peaks are located in the gene body (~14 %) or in regions within (~7 %) or beyond (~3 %) 500 bp upstream of the TSS. Given that the baseline of the input is significantly higher than that of GAF ChIP (GAF panel in Fig. 4a), the twofold enrichment appears to be a stringent criterion for selecting relevant genes for further analyses. The abundance and the position of GAF peaks strongly support these genes as *bona fide* physiological targets. Based on the number of putative GAF targets, our dataset is about 20–50 % larger than several earlier studies and similar to a recent study [30, 40, 51–53]. Notably, significant portions of the GAF targets were not identified in these studies (Additional file 1: Fig. S3). Using gene ontology (GO) analysis and Functional Annotation Clustering from DAVID, we found that our collection is enriched for genes involved in post-embryonic development, tissue morphogenesis, neural development, cell migration and sensory organ development [54, 55] (Fig. 3c). Thus, our study has revealed many new GAF targets involved in important developmental processes.

We next examined global gene expression profiles in WT and *Gaf* mutants. Triplicate RNA samples prepared from imaginal tissues of third-instar larvae were used to probe Agilent *Drosophila* Genome 2.0 arrays containing ~12,600 genes. We found that *Gaf* mutation significantly affected expression of 2912 genes (*p* < 0.05, >1.5-fold change). Among them, 15 genes were randomly chosen for verification by RT-qPCR. Nearly 70 % of selected genes showed similar changes (Additional file 1: Fig. S4A), confirming the validity of gene expression profiles. The vast majority (82 %) of affected genes showed reduced expression in *Gaf* mutants, supporting a positive role of GAF in global gene regulation (Fig. 3d). Unlike the strong enrichment of GAF peaks in many developmental processes, no specific process could be assigned to these affected genes (unpublished data). Further comparison with ChIP-seq data revealed that 269 genes contain GAF peaks with >twofold enrichment in the promoter region, indicating that they are directly regulated by GAF. As noted earlier, this number may be an underestimate, due to the large difference inherent in the calculation of ChIP and input samples. Importantly, about 80 % (218 genes) of them was down-regulated in *Gaf* mutants. In addition, they tend to cluster into functions related to neuronal development or morphogenesis (Fig. 3e). A recent study reported that 64 genes show altered expression in wing disks after GAF knockdown [56]. Using similar criteria for >twofold change, our study identified more than 2000 genes (Additional file 1: Fig. S4B), further validating our approach.

### GAF regulates promoter-proximal RNA-Pol

To determine the global effect of GAF on transcriptional processes, we examined genome-wide RNA-Pol distribution in both WT and mutant tissues by ChIP-seq. We used anti-Rpb3 antibody for an unbiased assessment of RNA-Pol distribution regardless of phosphorylation status [57]. By mapping about thirty million valid reads from WT or mutant samples, we identified about 2700 genes that satisfied the following criteria: false discovery rate (FDR) < 0.05 and *p* < 0.01 by Fisher’s exact test. The distribution of RNA-Pol and GAF in the 83B-C region is shown in Fig. 4a. RNA-Pol appears to be enriched in the promoter-proximal region of many genes within this section. In parallel, high levels of GAF are frequently found. Importantly, the abundance of promoter-proximal RNA-Pol of several GAF-target genes is reduced in mutants (average 40 % reduction in 52 % GAF-target genes).

To assess the global impact of GAF on RNA-Pol, we focused on GAF-target genes with more than twofold GAF enrichment in the promoter region. To avoid ambiguity, we selected genes based on criteria modified from a published article [7]: (1) single TSS; (2) no internal genes; and (3) at least 2 kb away from adjacent genes. A total of 365 genes were chosen for further analyses (Fisher’s exact test, *p* < 0.01) (Additional file 1: Fig. S5). For direct comparison, we first calculated RNA-Pol density by normalizing RNA-Pol reads at each nucleotide position with the total valid reads from WT or mutant samples, followed by summation of normalized values at each position. Subsequently, we summed up the value for the promoter-proximal region (region A, −100 to +150) and the gene body region (region B, +500 to the end of the gene) separately. Lastly, the relationship between RNA-Pol density and GAF occupancy was examined by plotting RNA-Pol density against the WT enrichment score of GAF for each gene.

As shown in Fig. 4b, e, GAF occupancy is significantly and positively correlated with the density of promoter-proximal RNA-Pol (Spearman’s rank correlation coefficient, *ρ* = 0.42, *p* < 1 × 10^−11^) in WT, whereas this correlation is almost completely lost in *Gaf* mutants (*ρ* = 0.01, *p* = 0.91). For the gene body, a modest negative correlation is observed between GAF occupancy and RNA-Pol density (*ρ* = −0.16, *p* = 0.002, Fig. 4c) in WT and is slightly affected in the *Gaf* mutants (*ρ* = −0.21, *p* = 3.7 × 10^−5^, Fig. 4f). We also adopted the pausing index (PI) to evaluate the genome-wide impact of *Gaf* mutation on transcriptional status [58]. To derive PI, the RNA-Pol values for regions A and B were used as numerators and denominators, respectively [9]. When PI was plotted against the enrichment score of GAF, a positive correlation between PI and GAF occupancy was also observed for the WT sample (*ρ* = 0.35, *p* = 1.1 × 10^−11^, Fig. 4d). Similarly, the correlation was reduced, albeit less evidently, in mutants (*ρ* = 0.15, *p* = 0.01, Fig. 4g). We attribute this mild effect to the larger reduction of denominators in mutants.

Among these targets, the extent of GAF occupancy appears to differ widely. Based on the normalized read at each nucleotide position, they range from a minimum of twofold over the input control to several hundred folds. To explore whether any group of genes might be more susceptible to GAF-mediated regulation, we divided these targets into four ranks based on the cumulative enrichment scores in the region from −500 to the TSS. Specifically, the normalized GAF reads in this region were added up for each gene and then used to calculate the enrichment over the input. The enrichment scores of these gene range from 2 to 5.9 (I, 92 genes), 6.1 to 10.0 (II, 91 genes), 10.0 to 17.2 (III, 91 genes), 17.5 to 301 (IV, 91 genes including 6 *Hsp70* genes). As expected from higher GAF occupancy, more extended GA repeats were found in ranks III and IV (Additional file 1: Fig. S6). Close inspection of GAF occupancy in these genes revealed that they differ from each other not only in their scores, but also in their overall distribution (Fig. 4h). For example, rank IV lacks a downstream GAF peak around +300 commonly found in other ranks, despite its prominent overall scores. Although GAF peaks are present in the upstream region of all targets, their positions appear to shift gradually from the TSS (~−10 in rank I) toward more upstream sites (~−100 in rank IV). Moreover, rank IV has relatively high levels of GAF occupancy up to the region around −500 (Fig. 4h).

Using box plots to represent the PI range for each rank, we found that higher ranks tended to show higher PIs, further reinforcing the relationship between GAF and paused RNA-Pol. Again, a more severe reduction of PIs was observed for higher ranks in mutants (Fig. 4i). We also examined the effect of *Gaf* mutation on RNA-Pol accumulation in the promoter-proximal region and gene body separately. Similarly, rank IV exhibited the most pronounced effect of RNA-Pol accumulation in the promoter-proximal region (Additional file 1: Fig. S7). These results strongly support a dose-dependent relationship between GAF occupancy and paused RNA-Pol. It is worth noting that only a small proportion of paused genes (~16.5 % of 1602 genes with PI > 4) identified at embryonic stages overlaps with our collection [9], reflecting highly dynamic regulation of pausing during development [59] (Additional file 1: Fig. S8).

### GAF affects the organization of upstream nucleosomes

**Fig. 5:**
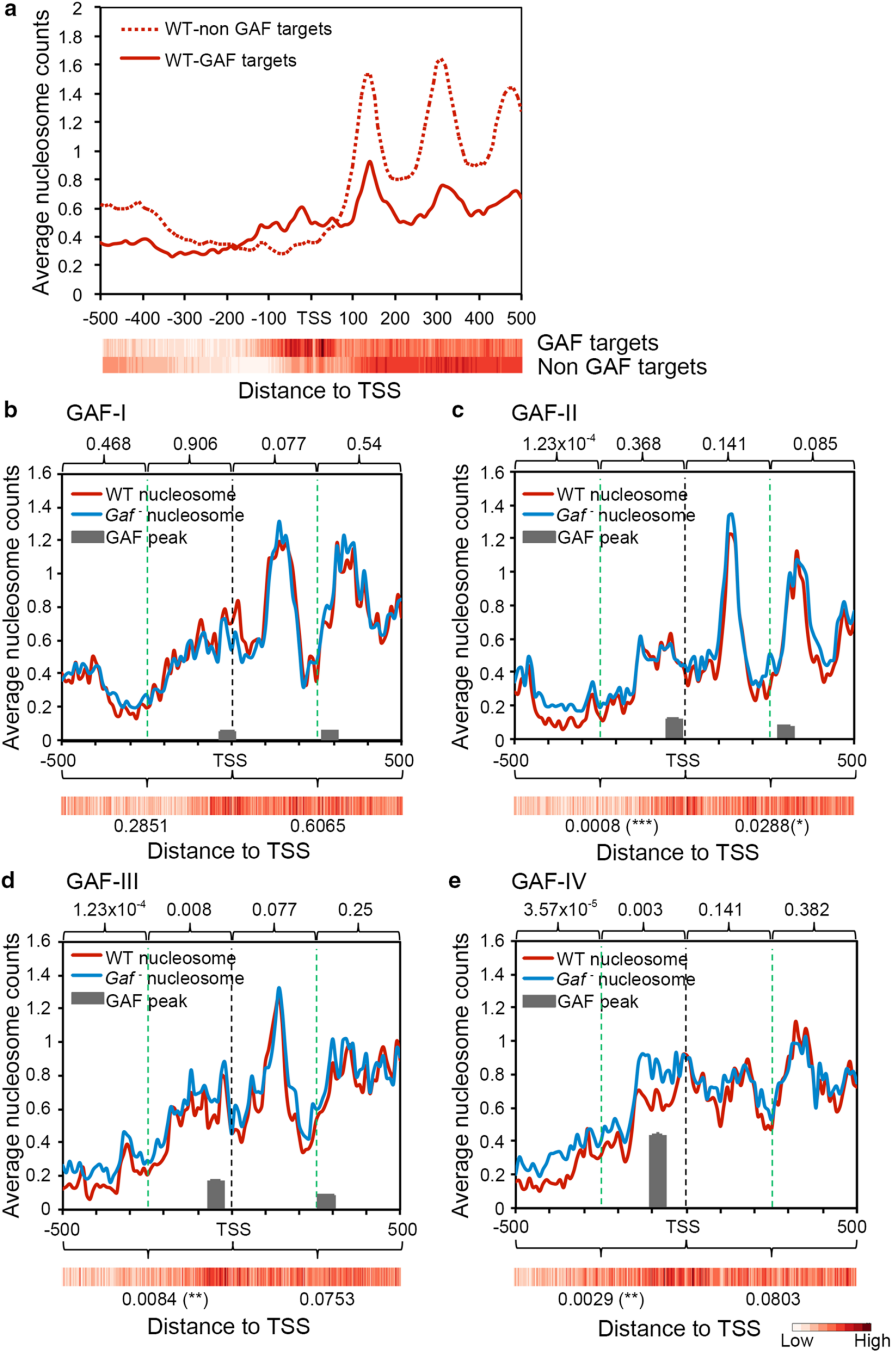
Selective effects of GAF on upstream nucleosomes. **a** Global nucleosome profiles. The overall nucleosome distribution of GAF targets (*n* = 1891) or non-targets (*n* = 13,774) in the WT genome is shown for the 1 kb region around TSS. To calculate the average nucleosome counts, the midpoint of MNase-resistant fragments was used to represent the position of each nucleosome. The average read at each nucleotide coordinate was summed after normalization. The frequency of SS-dinucleotides that favor nucleosome formation is shown by heat maps (*below*). **b**–**e** Alteration of nucleosome patterns in *Gaf* mutants. Nucleosome distribution within 1 kb of the promoter-proximal region in WT (*red*) and *Gaf* mutants (*blue*) is shown for different ranks of targets. The statistical differences between these two patterns were calculated by Kolmogorov–Smirnov tests after dividing the 1 kb region into either *four sections* (*above graphs*) or *two sections* (*below graphs*). The value and statistical significance of each section (*) are shown. The approximate location and height of GAF peaks are indicated in each graph (*gray bars*). The SS-dinucleotide distribution is also shown as a heat map for each rank

Recent studies have demonstrated a strong link between transcriptional pausing and the presence of nucleosomes around the TSS [23, 25]. To determine whether the nucleosome organization is involved in GAF-mediated transcription pausing, we examined genome-wide nucleosome profiles on WT and mutant chromatin by paired-end deep sequencing of chromatin fragments after micrococcal nuclease digestion (MNase-seq). Approximately 40 million reads for 120–180 bp fragments were selected, and the midpoint of each read was taken to represent a nucleosome count. The cumulative value of nucleosome counts at each nucleotide coordinate in the region from −500 to +500 of the TSS was then used to construct the nucleosome profile for the metagene. For the vast majority of genes without GAF occupancy in the promoter region (13,774 genes), a high level of ordered nucleosome array was observed in the downstream region, starting from the +1 nucleosome positioned around +130 (dash line in Fig. 5a). In contrast, less nucleosome counts were detected in the upstream region up to about −300, resembling the nucleosome-free region (NFR) found in many organisms [60, 61]. For 1891 GAF-target genes, a different profile was seen. In general, downstream nucleosome peaks were shallower and less distinct. Instead of the NFR, significant amounts of nucleosome counts were detected in the upstream region ranging from the TSS to −150 (solid line in Fig. 5a). To exclude the possibility that this difference might result from the much larger pool size of non-target genes used for the plotting, we constructed a nucleosome profile from 1900 non-targets randomly chosen from five chromosomal arms. No matter the difference in pool size, the patterns are almost indistinguishable for non-targets (Additional file 1: Fig. S9A).

Interestingly, these profiles appeared to correlate with the predicted nucleosome propensity based on the occurrence of SS-dinucleotides comprising GG, GC, CC and CG [62]. For example, the high level of downstream nucleosome arrays found in non-target genes coincided with high SS-dinucleotide contents. In contrast, the NFR was relatively devoid of such sequences. For GAF targets, however, the highest level of SS-dinucleotide content was found in sequences flanking the TSS. Thus, the WT nucleosome profile appears to partially reflect the intrinsic property of DNA sequences.

To determine the role of GAF in modulating nucleosome organization, we next examined how nucleosome profiles are altered in different ranks of GAF targets (Fig. 5b–e). Superimposition of WT nucleosome profiles with GAF peaks revealed a sharp +1 nucleosome peak flanked by GAF peaks in the three lower ranks of genes. In rank IV, no distinct nucleosome peak was found in this region. Despite these differences, none of these downstream nucleosome profiles were significantly affected by *Gaf* mutations. In the upstream region spanning the TSS, disordered nucleosome profiles were observed in all ranks. Notably, their nucleosome profiles seemed to extend more toward the upstream region for ranks I through IV, paralleling the presence of higher SS-dinucleotide content. The upstream nucleosome profile began to show significant increases from rank II in *Gaf* mutants. By dividing the upstream region into distal and proximal halves, we found that the increase in nucleosome counts was mainly located in the distal half of rank II (Kolmogorov–Smirnov test, *p* = 1.23 × 10^−4^). The increase expanded to the proximal half in rank III (*p* = 8 × 10^−3^) and became most pronounced in rank IV (*p* = 3 × 10^−3^). Thus, nucleosome organization is affected in a dose-dependent manner. It is also important to note that the upstream nucleosome profile of rank IV became more consistent with the high SS-dinucleotide content in *Gaf* mutants, suggesting that GAF’s main function is to alleviate the constraint imposed by nucleosomes and underlying sequences, particularly in the proximal half of the upstream region.

The correlation between increased nucleosome occupancy and reduced RNA-Pol strongly suggests that these events are related [23]. To determine whether reduced RNA-Pol is sufficient to cause the increase in nucleosome occupancy, we examined the nucleosome profile of non-target genes with significant reduction (>20 % reduction from WT, *n* = 210) of promoter-proximal RNA-Pol in *Gaf* mutant. Compared to the WT profile, no significant change was found in mutants (Additional file 1: Fig. S9B). We also examined the nucleosome profile around intergenic GAF peaks (*n* = 516). Interestingly, these regions showed substantial increases in nucleosome occupancy in mutants (Additional file 1: Fig. S9C). These observations strongly suggest that elevated nucleosome occupancy in *Gaf* mutants is not directly affected by adjacent RNA-Pol.

### GAF cooperates with other regulatory factors

Several motifs are frequently found in *Drosophila* core promoters [63]. Some appear to correlate with the occurrence of paused RNA-Pol or specific nucleosomal profiles [24, 64, 65]. We used MEME to survey the whole genome to determine their prevalence in GAF targets. Compared to non-targets, GAF targets showed significant enrichment for DPE (2.12 vs 3.60 %), INR (3.33 vs 5.98 %) and MTE (2.02 vs 4.92 %), but reduction for TATA boxes (6.29 vs 3.75 %). Interestingly, the enrichment for DPE, INR and MTE, as well as the reduction for TATA boxes, was further enhanced in rank IV (Table 1). In addition, the occurrence of Motif 1, 6, 7 and DRE was also significantly reduced in rank IV. These patterns are similar to those found in developmentally regulated genes [64].

**Table 1:** Frequency of various core promoter elements in non-target genes, GAF target genes and each GAF rank

Motif	Non-GAF targets	GAF targets	GAF-I	GAF-II	GAF-III	GAF-IV
DRE	9.05 %	9.89 %	20.65 %	12.09 %	18.68 %	3.30 %
Motif 1	13.32 %	15.18 %	23.91 %	19.78 %	9.89 %	9.89 %
Motif 5	5.67 %	6.24 %	8.70 %	6.59 %	4.40 %	6.59 %
Motif 6	15.72 %	14.17 %	17.39 %	16.48 %	12.09 %	7.69 %
Motif 7	11.70 %	13.38 %	21.74 %	20.88 %	9.89 %	6.59 %
DPE	2.12 %	3.60 %	1.09 %	1.10 %	5.49 %	7.69 %
INR	3.33 %	5.98 %	5.43 %	10.99 %	7.69 %	7.69 %
MTE	2.02 %	4.92 %	4.35 %	3.30 %	5.49 %	8.79 %
TATA box	6.29 %	3.75 %	6.52 %	4.40 %	2.20 %	1.10 %
Gene number	13,774	1891	92	91	91	91

Each element was screened by MEME with *p* value <0.005

**Fig. 6:**
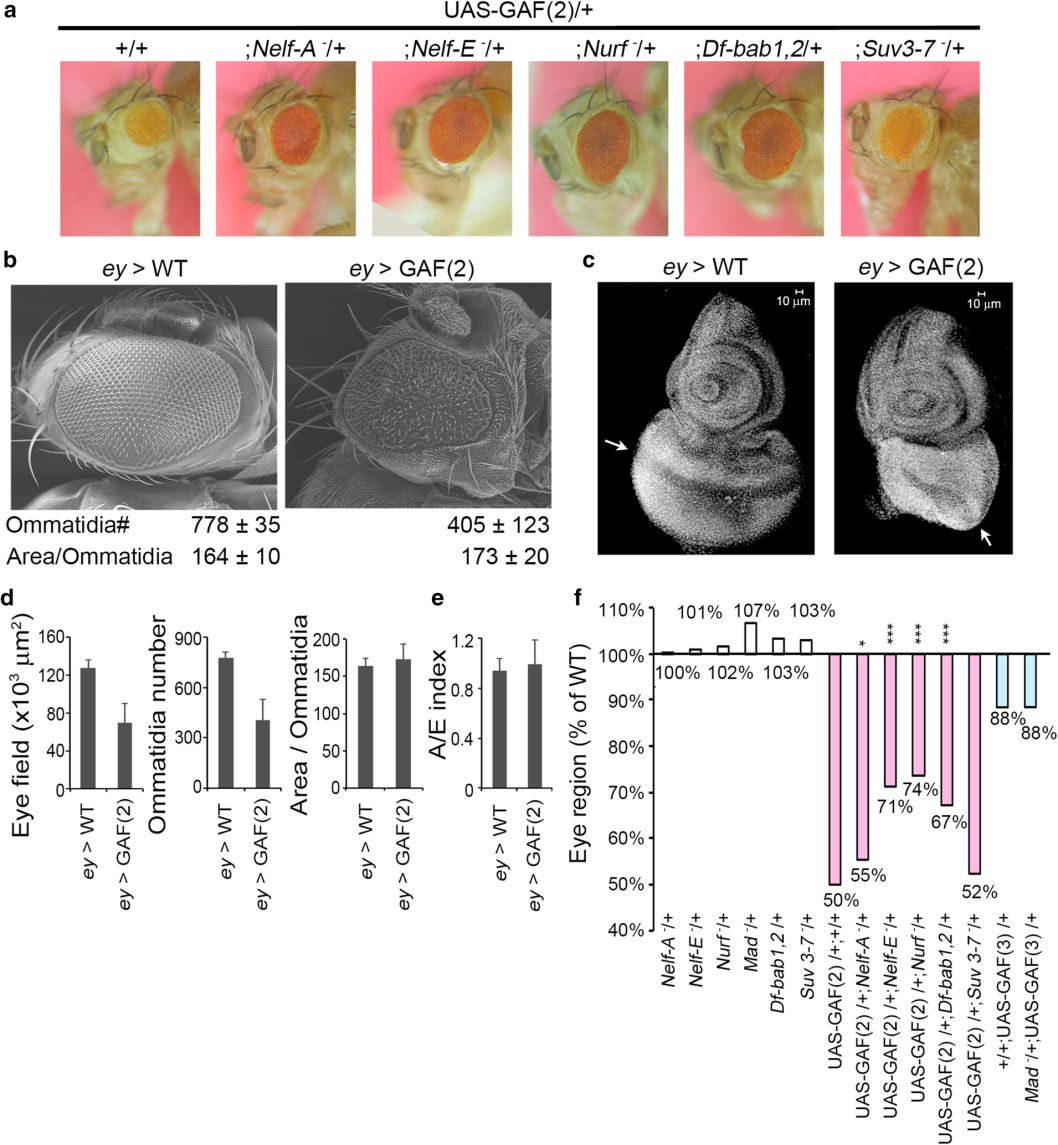
GAF-interacting factors involved in RNA-Pol pausing. **a** Genetic test for GAF-interacting factors. The small-eye phenotype induced by *ey* > GAF(2) was used to identify factors involved in GAF-mediated pausing. Each photograph represents the typical eye observed in WT and mutant backgrounds. **b** Detailed analysis of GAF-induced small-eye phenotype. The scanning electron microscopic graphs of typical eyes from *ey* > WT and *ey* > GAF(2) adults are shown. The total numbers of ommatidia in each eye field and the average area of each ommatidium were measured from these graphs (*ey* > WT, *n* = 12; *ey* > GAF(2), *n* = 22) and summarized below. **c** The effect of GAF over-expression in eye-antenna disks. Disks were stained by Hoechst 33258. The area posterior to the morphogenetic furrow (*arrow*) is severely reduced in *ey* > GAF(2) disks. **d** Quantitative measurement for adult eyes. The scanning electron microscopic graphs described in **b** were used to measure the eye field and ommatidia number separately. The average area per ommatidium was calculated from these values. **e** Quantitative measurement for larval imaginal disks. The nuclei density of antenna and eye disks from *ey* > WT and *ey* > GAF(2) animals was separately measured. The ratio of nuclei density between antenna and eye disks was given as A/E index. The details of the measurement are described in Additional file 1: Fig. S10A. **f** Effects of different mutations on small-eye phenotype. Measurement was based on the pixel number within eye perimeter in photographs taken under a dissection microscope. The average eye size was calculated from measurement of 50 eyes for each genotype. Statistical significance is indicated (*t* test, *p < 0.05; **p < 0.01; ***p < 0.001)

We next asked whether any other factors might cooperate with GAF in regulating transcriptional pausing. Since GAF over-expression could result in striking increases in GAF occupancy and paused RNA-Pol globally (Fig. 2b) and since there are multiple GAF motifs in eye development genes including *eyegone*, *twin of eyg*, *lozenge*, *seven*-*up* and *Bar* (data not shown), we reasoned that GAF over-expression in eye disks might lead to its increased occupancy on these promoters and subsequent pausing, providing a convenient genetic assay. Using *eyeless*-Gal4 to drive a UAS-GAF-519 transgene located on second chromosome (i.e., *ey* > GAF(2)) in eye imaginal tissues, we found that the size of the adult eyes was reduced by ~50 % in *ey* > GAF(2) flies (Fig. 6a, b). Detailed examination revealed that both eye field and ommatidia number were severely reduced (Fig. 6b, d). However, the average space of each ommatidium remained similar for both *ey* > WT and *ey* > GAF(2) adults. We further examined the structure of larval eye disks. As expected, the size of eye disks of third-instar larvae was substantially reduced (Fig. 6c). Specifically, the region corresponding to undifferentiated cells located posterior to the morphogenetic furrow showed impaired development. We also compared the nuclei density of antenna disks with that of posterior eye disks (A/E index, Fig. 6e and Additional file 1: Fig. S10A). Again, similar values were found for *ey* > WT and *ey* > GAF(2) disks. Thus, GAF over-expression appears to mainly affect the number of eye cells.

Previous studies have shown that NURF—an ATP-dependent nucleosome remodeling factor—may interact directly with GAF to re-organize nucleosomal spacing [38]. In a *Nurf* mutant background, eye size was substantially restored (Fig. 6a, f and Additional file 1: Fig. S10B), indicating that *Nurf* is indeed involved in GAF-mediated regulation. We further tested the involvement of other factors known to facilitate RNA-Pol pausing directly [21]. Specifically, two components of NELF complex were examined. Interestingly, the small-eye phenotype was relieved moderately by a *Nelf*-*A* mutation and substantially by a *Nelf*-*E* mutation. However, the mutation of *Su(var)3*-*7*—a suppressor of heterochromatin-mediated silencing [66]—did not produce significant effects.

Furthermore, we used the MEME program to search potential binding motifs in the region from −1000 to +500 of GAF-target genes. These motifs were then analyzed by the TOMTOM program to identify corresponding transcription factors [50], followed by verification with the FlyTF database containing 753 validated site-specific transcription factors [67]. Relevant factors and their binding motifs are summarized in Table 2. Consistent with our ranking, most significant GAF motifs were found in ranks III and IV (*E* values <5.2 × 10^−37^ or 6.3 × 10^−103^). Interestingly, motifs for BAB1 (*E* value <2.6 × 10^−21^) and MAD (E value <1.5 × 10^−10^) were identified in rank IV only. Again, we tested their physiological relevance using the GAF-induced eye phenotype assay. The eye defects were substantially relieved in *bab1* (Fig. 6a, f), suggesting the functional involvement of BAB1 in GAF-mediated pausing. In contrast, no obvious effect was observed in *Mad* mutant. However, due to the weaker eye phenotype induced by UAS-GAF located on third chromosome (i.e., UAS-GAF(3)), the role of MAD is less certain (Fig. 6f and Additional file 1: Fig. S10B).

**Table 2:** Summary of potential transcription factors in each GAF rank

GAF ranks	TFs	MEME	TOMTOM	
		*E* values	*p* values	*q* values
GAF-I	SR	3.6 × 10^−12^	5.9 × 10^−6^	4.8 × 10^−3^
	KLU		7.2 × 10^−6^	5.8 × 10^−3^
	FRU		1.2 × 10^−5^	9.4 × 10^−3^
	RN		4.1 × 10^−5^	3.3 × 10^−2^
GAF-II	JIM	3 × 10^−32^	3.4 × 10^−7^	2.7 × 10^−4^
	RN		4.2 × 10^−5^	3.4 × 10^−2^
	JIGR1		4.9 × 10^−5^	4 × 10^−2^
GAF-III	GAF	5.2 × 10^−37^	3.3 × 10^−6^	2.7 × 10^−3^
	JIM	2.3 × 10^−19^	2.1 × 10^−6^	1.7 × 10^−3^
	JIGR1		1.4 × 10^−5^	1.1 × 10^−2^
	RN		5 × 10^−5^	4 × 10^−2^
GAF-IV	GAF	6.3 × 10^−103^	2.2 × 10^−6^	1.8 × 10^−3^
	BAB1	2.6 × 10^−21^	6.5 × 10^−5^	5.3 × 10^−2^
	MAD	1.5 × 10^−10^	1.3 × 10^−5^	1.1 × 10^−2^
	JIM	1.2 × 10^−4^	6.5 × 10^−6^	5.3 × 10^−3^
	RN		5.6 × 10^−5^	4.5 × 10^−2^

The *E* value indicates the statistical significance of the motif used by MEME. The *p* value indicates the probability of motif match by random chance. The *q* value indicates the minimal false discovery rate

## Discussion

In this report, we used several approaches to address the role of GAF in transcriptional regulation in broad tissue contexts. Our results support that GAF acts as a global activator and is required to maintain proper level of paused RNA-Pol and organization of upstream nucleosomes.

As expected from prevalent GAF occupancy in the genome, expression of ~20 % of coding genes (2912 genes) is affected by *Gaf* mutation. Based on its occupancy near the TSS, we estimate that at least 9 % of them are directly regulated by GAF. Interestingly, about 15 % of these direct targets (40 genes) are listed in FlyTF as putative transcriptional factors (Additional file 2: Table S1), of which nearly half have previously been validated experimentally and are known to carry out important developmental functions such as embryonic segment formation and cell fate determination. Conceivably, these transcriptional factors could further control the expression of a much larger spectrum of downstream genes that lack direct GAF binding. Either directly or indirectly, the vast majority (~80 %) of these genes are down-regulated in *Gaf* mutants. These results demonstrate that GAF functions primarily as a global transcriptional activator. It is worth noting that many of these developmental regulators were not identified in previous studies [26, 51]. Therefore, our results significantly expand the global function of GAF, particularly in the developmental processes.

For the inducible *Hsp70* gene, we demonstrate that GAF affects transcription primarily at post-initiation steps. Both cytogenetic and molecular analyses of the *Hsp70* gene showed that the levels of Ser-5p and Ser-2p, but not Hypo-p, are substantially affected in *Gaf* mutants following heat induction. Since phosphorylation of RNA-Pol occurs after transcriptional initiation, these results clearly indicate that GAF exerts its effects at post-initiation steps. Our studies also revealed reciprocal changes for Ser-5p and Ser-2p in *Gaf* mutants; accompanying the large decrease in promoter-proximal Ser-5p signal, Ser-2p showed a substantial increase toward the distal end of *Hsp70*. Given the obligatory order for the appearance of Ser-5p and Ser-2p during transcription, these observations strongly suggest that the reduction of Ser-5p signal represents the primary effect of *Gaf* mutation. The increase in Ser2-p seems to be at odds with the observation that the majority of genes are down-regulated in *Gaf* mutants. Since GAF can physically interact with NELF and since active transcription of *Hsp70* occurs when NELF is dissociated from RNA-Pol [21, 68], it is plausible that less NELF is recruited to *Hsp70* promoter in *Gaf* mutant, resulting in less efficient withholding of paused RNA-Pol. In addition, we note that *Hsp70* expression can still be induced by the potent activator HSF during heat shock from a transgene lacking GAF binding sites [69]. Thus, *Hsp70* may represent an unusual case in which multiple factors are critically involved in its activation. GAF knockdown in cultured cells has recently been reported to affect the accumulation of total RNA-Pol only in promoter-proximal region [40]. Since the individual contribution of Ser-5p and Ser-2p could not be distinguished in that assay and since we show that there is a reciprocal relationship between these two isoforms particularly in distal regions, it is very likely that the effects of GAF depletion were not fully evaluated.

Our results further show that the effect of GAF on Ser-5p is not unique to *Hsp70*. We found that *Gaf* mutation results in reduced Ser-5p, but not Hypo-p or Ser-2p, on polytene chromosomes, suggesting that GAF can modulate the level of Ser-5p globally. This is substantiated by our analyses of the genome-wide distribution of RNA-Pol. In WT samples, GAF occupancy in the upstream region is positively correlated only with the RNA-Pol density in the promoter-proximal region or with PI. These results clearly reveal a dose-dependent relationship between paused RNA-Pol and GAF. Importantly, this positive correlation was lost in *Gaf* mutants, indicating a direct role of GAF in RNA-Pol pausing. Contrary to the reduction of paused RNA-Pol in *Gaf* mutants, GAF over-expression results in an overall increase in Ser-5p, but not other isoforms. These complementary results further strengthen the conclusion that GAF is critical for transcription pausing.

Consistent with earlier studies [26, 68], our analyses of genome-wide nucleosome profiling revealed remarkable differences between GAF-target and non-target genes. Much lower levels of the nucleosome array were found in the transcribed region of GAF-target genes, indicating that paused genes contain less downstream nucleosomes. Although somewhat anti-intuitive, these patterns are consistent with recent studies showing a negative correlation between PI and downstream nucleosome occupancy [23]. Apparently, these observations do not support a general notion that downstream nucleosomes act directly as a barrier for RNA-Pol, triggering its pausing. Interestingly, these profiles appear to coincide closely with the SS-dinucleotide content predicted to favor nucleosome occupancy, suggesting a close relationship between them.

In contrast to the downstream region, GAF-target genes contain more nucleosomes around the TSS and immediately upstream region than non-target genes. Overall, these patterns are consistent with the SS-dinucleotide content of the upstream region. However, considering the strong enrichment of SS-dinucleotide content in GAF targets, the increased nucleosome levels seem to be somewhat modest. It is also puzzling that nucleosomal profiles of different ranks of target genes appear to be largely indistinguishable, despite there being more extended SS-dinucleotide sequences in higher ranks. Nevertheless, the levels of nucleosomes were selectively increased in upstream regions from the TSS up to −500 for rank III and more so for rank IV in *Gaf* mutants, resulting in closer matches to predicted nucleosome profiles. Thus, the upstream sequences of these genes may intrinsically favor higher levels of nucleosomes, potentially producing stronger physical constraints for the loading and assembly of transcriptional machinery. Apparently, the presence of high levels of GAF relieves such constraints by reducing the amount of nucleosomes and consequently makes promoters more accessible. Like RNA-Pol pausing, the modulation of nucleosome profiles by GAF is highly dependent upon the degree of occupancy. This dosage-dependent effect strongly supports a direct role of GAF. Thus, caution should be taken when making inferences about GAF’s role in regulation based on the GAF motif alone.

Since GAF can recruit NURF to remodel nucleosome organization in vitro [38] and since our genetic studies showed that NURF is involved in GAF-mediated regulation, we suggest that GAF may act through NURF to remodel upstream nucleosomes and subsequently facilitate the loading and assembly of factors involved in regulation and general transcription. Based on the observation that a reduction of paused RNA-Pol is correlated with elevated upstream nucleosomes [23, 59], an alternative might suggest a different sequence of events. However, this possibility is not supported by our observations that nucleosome occupancy is not affected in non-target genes with significantly reduced RNA-Pol and the nucleosome occupancy is elevated in intergenic regions in *Gaf* mutants.

The role of GAF in RNA-Pol pausing and nucleosome remodeling has recently been reported, based on similar studies of a cultured cell line [40]. While our results on RNA-Pol pausing are quite similar, the effects on nucleosomal remodeling are somewhat different. In our study, nucleosome perturbation is found only in the upstream region where maximal GAF occupancy is located, while the earlier study has shown that the effect is exerted on more extended region including TSS and further downstream. Since our collections of GAF targets differ by ~50 %, this difference may reflect the selection of different gene pools. It is also worth noting that multiple tissues are used in our study. Thus, the effect we have observed may represent a more general scheme in living organisms.

**Fig. 7:**
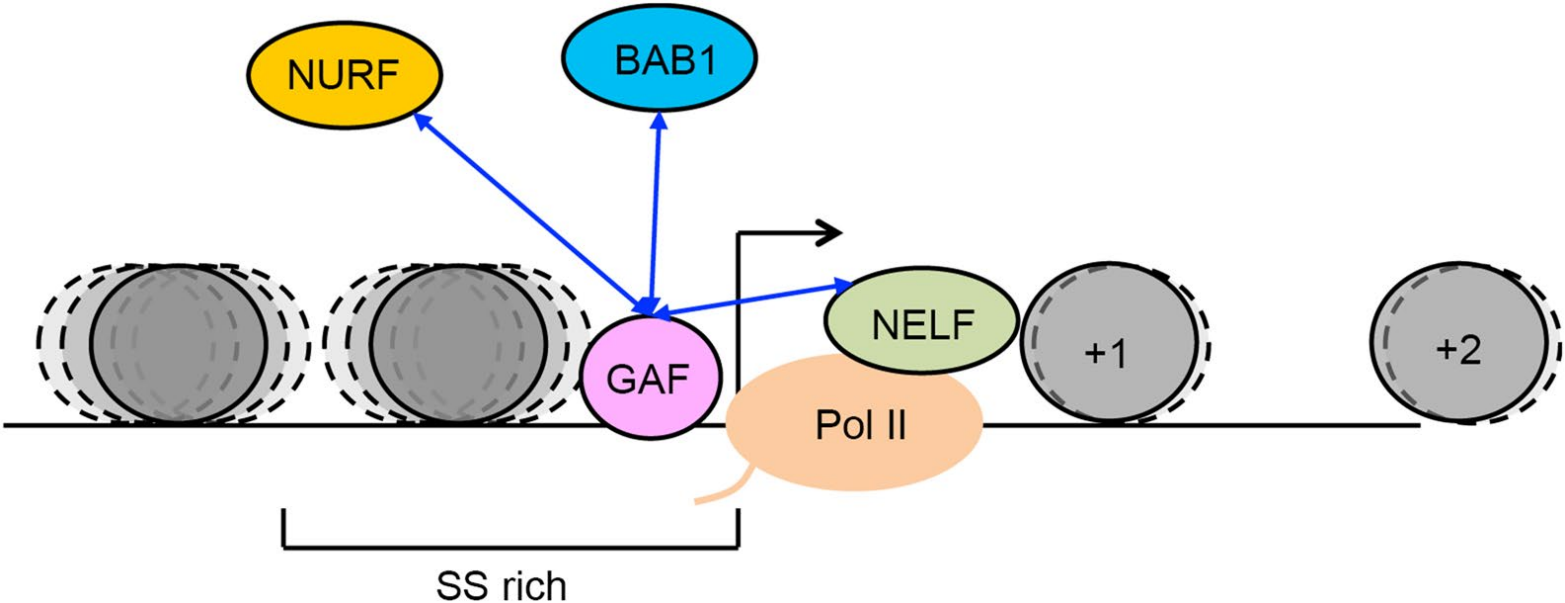
Proposed model for GAF-mediated transcriptional pausing. Genes with high SS-dinucleotide contents around and upstream of the TSS are occupied by much higher levels of upstream nucleosomes (*dark circle*) than others. This organization prevents the loading and assembly of the transcriptional complex. The occupancy of GAF results in the recruitment and cooperative interactions with NURF, NELF and BAB1 to facilitate local nucleosome remodeling and provide accessible space for the assembly of the transcriptional complex and subsequently triggers its transition to the paused state

In addition to NURF, our study shows that NELF and BAB1 are physiologically relevant to GAF’s function. Since NELF can physically interact with GAF and RNA-Pol, GAF may also regulate pausing through recruitment of NELF. However, comparison of the available data suggests that GAF targets overlap only partially with those of NELF or NURF [30, 70] (Additional file 1: Fig. S11). Therefore, GAF may act cooperatively with individual or multiple factors, depending upon specific targets. Furthermore, earlier studies suggest that BAB1 and GAF share a common interacting protein, TAF3, a component of TFIID essential for the assembly of the pre-initiation complex [71, 72]. Our findings that BAB1 binding sites are enriched specifically in rank IV genes and that there is a strong genetic interaction between *bab1* and *Gaf* mutants support cooperation between GAF and BAB1 in controlling transcriptional pausing. These results clearly show that the upstream region of GAF targets is intrinsically prone to nucleosome assembly, resulting in promoter occlusion. By its ability to trigger nucleosome re-organization, GAF may facilitate the promoter accessibility, assembly of the basal transcriptional machinery and its transition into initiation (Fig. 7). Subsequently, the activity of pausing factors recruited by GAF may arrest RNA-Pol at the pausing state, awaiting developmental or environmental cues. Thus, GAF appears to act as a central player of this regulatory circuitry.

## Conclusion

We present cytogenetic, transcriptomic, chromatin landscaping and nucleosome profiling studies on transcriptional pausing of *Hsp70* and whole genome to understand the role of a *Drosophila* upstream binding factor, GAF. Our studies show that depletion of GAF results in global reduction of gene expression, reduced transcriptional pausing of genes that normally have high levels of GAF occupancy. These genes appear to be enriched for sequences that favor nucleosome occupancy in the upstream region. The binding of GAF in these genes and its cooperative interactions with factors involved in nucleosome remodeling and RNA-Pol pausing relieve constraints imposed by nucleosome occupancy, enabling these promoters more permissive for the progression of early transcriptional processes and poised for subsequent induction. Our results support a pivotal role of GAF in a regulatory circuitry. It provides the first mechanistic illustration for regulation of transcriptional pausing by an upstream factor in an animal model.

## Methods

### Fly preparation

All flies were raised in standard media at 25 °C. *Gaf*^*13C#3*^ carrying *st cu sr e* ca markers was derived from recombination between *Trl*^*13C*^ [36] and a standard *ru cu* ca strain. *Gaf*^*DH34*^ was generated from *EP*^*3184*^ (Szeged Stock Centre) by immobilization of the P-element. *Nelf*-*A*^*KG09483*^, *Nelf*-*E*^*EY07065*^, *Df(3L)bab*^*Ar07*^, *Mad*^*12*^ and *ru cu* ca were obtained from Bloomington Stock Centre. *w; P*{*GawB*}*Cdk9*^*NP0727*^*/CyO* was from Drosophila Genetic Resource Center. *E(bx)*^*Nurf301*−*9*^ and *Su(var)3*-*7*^*14*^ were obtained from P. Badenhorst and P. Spierer, respectively.

### Antibody and immunoblotting

GST-GAF-N was constructed by inserting a GAF N-terminal fragment (1–1089 nucleotides of ORF) into pGST5X-1 (Novagen). Bacterial fusion protein induced in strain BL21 (DE3) was gel-purified and used for immunization in rabbits by standard protocols [73]. After pre-absorption with GST extracts, the antibody was affinity-purified, using GST-GAF-M fusion protein containing the middle part of GAF (253–1352 nucleotides of ORF). For immunoblotting, imaginal tissues from WT and *Gaf* mutant larvae were lysed in SDS–urea buffer [73]. Extracts were separated on 10 % SDS-PAGE, blotted onto nitrocellulose filters and detected by antibodies against GAF, α-tubulin (Sigma, T9026), RNA-Pol including ARNA3 (Merck Millipore, CBL221), CTD Hypo-p (8wg16) (Covance, MMS-126R), Ser-5p (H14) (Covance, MMS-134R), Ser-2p (H5) (Covance, MMS-129R).

### Polytene chromosome staining

Three pairs of salivary glands were isolated from third-instar larvae raised at 25 °C or treated for 30 min at 37 °C before dissection. Polytene chromosomes prepared from squashed glands were stained according to previously described procedures [74, 75]. Primary antibodies were used at the following dilutions: GAF, 1:500; BEAF-32, 1:50 (Developmental Studies Hybridoma Bank); RNA-Pol 8wg16, H14, H5, all 1:20; Rpb3, 1:100 (a gift from Dr. J. T. Lis). Secondary antibodies were used at 1:100 dilution for Cy5-conjugated goat anti-rabbit IgG (Jackson ImmunoResearch, 111-175-144), rhodamine-conjugated goat anti-mouse IgM (Jackson ImmunoResearch, 715-595-140), Alexa 488 goat anti-mouse IgG1 (Molecular Probes, A21202), Alexa 633 goat anti-mouse IgG2a antibodies (Molecular Probes, A21105). DNA was stained with Hoechst 33258 (0.25 mg/ml) (Polysciences, 09460). The fluorescent staining was detected by Zeiss LSM 510 confocal microscope.

### ChIP analysis for *Hsp70*

Thirty WT or mutant third-instar larvae were dissected in *Drosophila* M3 medium (Sigma); anterior-most parts containing imaginal disks, nerve cords and salivary glands (referred to as imaginal tissues throughout) were collected. For heat-shock samples, tissue samples were processed according to published procedures [76] and then incubated at 37 °C for 5 min. Tissues were fixed for 20 min in 1 % paraformaldehyde and quenched by 0.125 M glycine. After three washes in TBS (10 mM Tris–HCl, pH 8.0 and 150 mM NaCl), fixed tissues were suspended in 0.5 ml of lysis buffer (Upstate) and then sheared by Bioruptor (Diagenode). A 0.1-ml aliquot was used for each immunoprecipitation assay with different antibodies or pre-immune serum according to the vendor’s protocol (Upstate). The amount of antibodies used in these assays was as follows: Ser-5p, 15 μL; Ser-2p, 25 μL; Hypo-p, 5 μL; GAF, 2.5 μL. Two percent of immunoprecipitated DNA or input DNA was subjected to qPCRs for 35 cycles. PCR primers are listed in Additional file 2: Tables S2, 3.

### Microarray analyses

Total RNA was extracted from imaginal tissues of WT or mutant third-instar larvae by TRIzol (Invitrogen), treated with RNase-free DNase I (Worthington) and purified by RNeasy kit (Qiagen) according to the vendors’ protocols. Microarray analysis was conducted by our in-house Microarray Core Facility (http://www.imb.sinica.edu.tw/mdarray/index.html). Briefly, cDNA was synthesized from 15 μg RNA and labeled with Alexa Fluor 555 (WT) or Alexa Fluor 647 (mutant) using a SuperScript Indirect cDNA Labeling System (Invitrogen). Labeled probes prepared from three samples were independently hybridized to a Drosophila Gene Expression Microarray (Agilent 4 × 44 K) containing 37,536 oligonucleotide probes, according to the manufacturer’s protocol. Microarrays were scanned on an Agilent G2565CA scanner and quantified using Feature Extraction software (FE version 10.5.1.1.1) for background subtraction and LOWESS normalization. GeneSpring GX11.5 analysis software (Agilent) was used for further analysis. Average values from triplicate sets were filtered on a Volcano plot, and an entity list was collected from probes with significant expression in WT samples (signal intensity > 100, *p* < 0.05).

### ChIP-sequencing

Chromatin samples from WT and mutant larvae were prepared as described above. Immunoprecipitation was performed with affinity-purified antibodies against GAF or Rpb3. ChIP and input DNA samples were processed using an Illumina ChIP-Seq DNA Sample Prep kit (Illumina). After ligation to adaptors, DNA samples were amplified by PCR without size selections. Sequencing was performed on an Illumina Genome Analyzer IIx for 40 bp read length by the Biofuel High Throughput Sequencing Core Facility (Institute of Biodiversity Research Center, Academia Sinica, Taiwan).

### MNase sequencing

MNase-digested chromatin was prepared as described previously with some modifications [23, 77, 78]. After fixation, imaginal tissues were resuspended in 1 ml buffer A (300 mM sucrose, 2 mM Mg acetate, 3 mM CaCl_2_, 10 mM Tris pH 8, 0.1 % Triton X-100 and 0.5 mM DTT), for 60 larvae and homogenized in a Dounce homogenizer (tight pestle, Wheaton). Nuclei were collected by centrifugation at 4 °C, 720 *g* for 5 min, washed by buffer A and washed in buffer D (50 mM Tris–HCl pH 8, 25 % glycerol, 5 mM Mg acetate, 0.1 mM EDTA and 5 mM DTT). Nuclei were resuspended in buffer MN (15 mM Tris–HCl pH 8.5, 60 mM KCl, 15 mM NaCl, 0.5 mM DTT, 0.25 M sucrose and 3 mM CaCl_2_). Two hundred microliter of chromatin was digested with 20U of MNase (Worthington) for 3 h at 25 °C. Gel-purified DNA fragments ranging from 100 to 200 bp were then subjected to pair-end sequencing by an Illumina Genome Analyzer IIx.

Details of bioinformatic analyses are presented in Additional file 3.

## Additional files

10.1186/s13072-016-0082-4 **Figure S1.** Analyses of RNA-Pol in Gaf mutants. **Figure S2.** RNA-Pol Hypo-p and Ser-2p are not affected by GAF on polytene chromosomes. **Figure S3.** Comparison of GAF target genes identified in different reports. **Figure S4.** Verification of gene expression by microarray studies. **Figure S5.** Procedures of statistical analyses of GAF and RNA-Pol ChIP-seq data. **Figure S6.** The abundance of different GAF binding motifs among targets. **Figure S7.** Reduction promoterproximal RNA-Pol in the Gaf mutant. **Figure S8.** Comparison of paused genes identified in different reports. **Figure S9.** Comparion of nucleosome profiles. **Figure S10.** Analyses of GAF’s effects on eye development. **Figure S11.** Comparison of target genes for GAF, NELF and NURF.
10.1186/s13072-016-0082-4 **Table S1.** Putative transcription factors identified in GAF target genes. **Table S2.** PCR primers used for ChIP analysis of *Hsp70*. **Table S3.** Primers used for RT-qPCR.
10.1186/s13072-016-0082-4 Supplementary Methods.

## Abbreviations

GAF: GAGA factor
RNA-Pol: RNA polymerase II
*Hsp70*: *heat shock 70*
MNase: micrococcal nuclease
NURF: nucleosome remodeling factor
NELF: negative elongation factor
bab1: *bric a brac 1*
Hypo-p: hypo-phosphorylated serine residues on C-terminal heptad repeats of Rpb1
Ser-5p: hyper-phosphorylated serine 5 residues on C-terminal heptad repeats of Rpb1
Ser-2p: hyper-phosphorylated serine 2 residues on C-terminal heptad repeats of Rpb1
DSIF: DRB sensitivity-inducing factor
FACT: facilitates chromatin transcription
*Trl*: *trithorax*-*like*
BEAF-32: boundary element-associated factor of 32kD
*dpp*: *decapentaplegic*
PICS program: Probabilistic inference for ChIP-Seq
MEME: Multiple EM for Motif Elicitation
DAVID: Database for Annotation, Visualization and Integrated Discovery
P-TEFb: positive transcription elongation factor b
*Mad*: *mothers against dpp*
*Su(var)3*-*7*: suppressor of variegation 3–7

## References

[1] Fuda NJ, Ardehali MB, Lis JT (2009). Defining mechanisms that regulate RNA polymerase II transcription in vivo. Nature.

[2] Thomas MC, Chiang C-M (2006). The general transcription machinery and general cofactors. Crit Rev Biochem Mol Biol.

[3] Rougvie AE, Lis JT (1990). Postinitiation transcriptional control in Drosophila melanogaster. Mol Cell Biol.

[4] Krumm A, Meulia T, Brunvand M, Groudine M (1992). The block to transcriptional elongation within the human c-myc gene is determined in the promoter-proximal region. Genes Dev.

[5] Plet A, Eick D, Blanchard JM (1995). Elongation and premature termination of transcripts initiated from c-fos and c-myc promoters show dissimilar patterns. Oncogene.

[6] Strobl LJ, Eick D (1992). Hold back of RNA polymerase II at the transcription start site mediates down-regulation of c-myc in vivo. EMBO J.

[7] Nechaev S, Fargo DC, dos Santos G, Liu L, Gao Y, Adelman K (2010). Global analysis of short RNAs reveals widespread promoter-proximal stalling and arrest of Pol II in Drosophila. Science.

[8] Guenther MG, Levine SS, Boyer LA, Jaenisch R, Young RA (2007). A chromatin landmark and transcription initiation at most promoters in human cells. Cell.

[9] Zeitlinger J, Stark A, Kellis M, Hong J-W, Nechaev S, Adelman K, Levine M, Young RA (2007). RNA polymerase stalling at developmental control genes in the Drosophila melanogaster embryo. Nat Genet.

[10] Min IM, Waterfall JJ, Core LJ, Munroe RJ, Schimenti J, Lis JT (2011). Regulating RNA polymerase pausing and transcription elongation in embryonic stem cells. Genes Dev.

[11] Kim TH, Barrera LO, Zheng M, Qu C, Singer MA, Richmond TA, Wu Y, Green RD, Ren B (2005). A high-resolution map of active promoters in the human genome. Nature.

[12] Levine M (2011). Paused RNA polymerase II as a developmental checkpoint. Cell.

[13] Adelman K, Lis JT (2012). Promoter-proximal pausing of RNA polymerase II: emerging roles in metazoans. Nat Rev Genet.

[14] Zhou Q, Li T, Price DH (2012). RNA polymerase II elongation control. Annu Rev Biochem.

[15] Phatnani HP, Greenleaf AL (2006). Phosphorylation and functions of the RNA polymerase II CTD. Genes Dev.

[16] Yamaguchi Y, Takagi T, Wada T, Yano K, Furuya A, Sugimoto S, Hasegawa J, Handa H (1999). NELF, a multisubunit complex containing RD, cooperates with DSIF to repress RNA polymerase II elongation. Cell.

[17] Wada T, Takagi T, Yamaguchi Y, Ferdous A, Imai T, Hirose S, Sugimoto S, Yano K, Hartzog GA, Winston F (1998). DSIF, a novel transcription elongation factor that regulates RNA polymerase II processivity, is composed of human Spt4 and Spt5 homologs. Genes Dev.

[18] Cheng B, Price DH (2007). Properties of RNA polymerase II elongation complexes before and after the P-TEFb-mediated transition into productive elongation. J Biol Chem.

[19] Price DH (2000). P-TEFb, a cyclin-dependent kinase controlling elongation by RNA polymerase II. Mol Cell Biol.

[20] Kwak H, Lis JT (2013). Control of transcriptional elongation. Annu Rev Genet.

[21] Wu CH, Yamaguchi Y, Benjamin LR, Horvat-Gordon M, Washinsky J, Enerly E, Larsson J, Lambertsson A, Handa H, Gilmour D (2003). NELF and DSIF cause promoter proximal pausing on the hsp70 promoter in Drosophila. Genes Dev.

[22] Hendrix DA, Hong J-W, Zeitlinger J, Rokhsar DS, Levine MS (2008). Promoter elements associated with RNA Pol II stalling in the Drosophila embryo. Proc Natl Acad Sci.

[23] Gilchrist DA, Dos Santos G, Fargo DC, Xie B, Gao Y, Li L, Adelman K (2010). Pausing of RNA polymerase II disrupts DNA-specified nucleosome organization to enable precise gene regulation. Cell.

[24] Rach EA, Winter DR, Benjamin AM, Corcoran DL, Ni T, Zhu J, Ohler U (2011). Transcription initiation patterns indicate divergent strategies for gene regulation at the chromatin level. PLoS Genet.

[25] Mavrich TN, Jiang C, Ioshikhes IP, Li X, Venters BJ, Zanton SJ, Tomsho LP, Qi J, Glaser RL, Schuster SC (2008). Nucleosome organization in the Drosophila genome. Nature.

[26] Li J, Gilmour DS (2013). Distinct mechanisms of transcriptional pausing orchestrated by GAGA factor and M1BP, a novel transcription factor. EMBO J.

[27] Lehmann M, Siegmund T, Lintermann KG, Korge G (1998). The pipsqueak protein of Drosophila melanogaster binds to GAGA sequences through a novel DNA-binding domain. J Biol Chem.

[28] Soeller WC, Oh CE, Kornberg TB (1993). Isolation of cDNAs encoding the Drosophila GAGA transcription factor. Mol Cell Biol.

[29] Gilchrist DA, Nechaev S, Lee C, Ghosh SKB, Collins JB, Li L, Gilmour DS, Adelman K (2008). NELF-mediated stalling of Pol II can enhance gene expression by blocking promoter-proximal nucleosome assembly. Genes Dev.

[30] Lee C, Li X, Hechmer A, Eisen M, Biggin MD, Venters BJ, Jiang C, Li J, Pugh BF, Gilmour DS (2008). NELF and GAGA factor are linked to promoter-proximal pausing at many genes in Drosophila. Mol Cell Biol.

[31] Benyajati C, Mueller L, Xu N, Pappano M, Gao J, Mosammaparast M, Conklin D, Granok H, Craig C, Elgin S (1997). Multiple isoforms of GAGA factor, a critical component of chromatin structure. Nucleic Acids Res.

[32] Wilkins RC, Lis JT (1997). Dynamics of potentiation and activation: GAGA factor and its role in heat shock gene regulation. Nucl Acids Res.

[33] Greenberg AJ, Schedl P (2001). GAGA factor isoforms have distinct but overlapping functions in vivo. Mol Cell Biol.

[34] Kerrigan LA, Croston GE, Lira LM, Kadonaga JT (1991). Sequence-specific transcriptional antirepression of the Drosophila Kruppel gene by the GAGA factor. J Biol Chem.

[35] Biggin MD, Tjian R (1988). Transcription factors that activate the Ultrabithorax promoter in developmentally staged extracts. Cell.

[36] Farkas G, Gausz J, Galloni M, Reuter G, Gyurkovics H, Karch F (1994). The Trithorax-like gene encodes the Drosophila GAGA factor. Nature.

[37] Adkins NL, Hagerman TA, Georgel P (2006). GAGA protein: a multi-faceted transcription factor. Biochem Cell Biol.

[38] Tsukiyama T, Wu C (1995). Purification and properties of an ATP-dependent nucleosome remodeling factor. Cell.

[39] Shimojima T, Okada M, Nakayama T, Ueda H, Okawa K, Iwamatsu A, Handa H, Hirose S (2003). Drosophila FACT contributes to Hox gene expression through physical and functional interactions with GAGA factor. Genes Dev.

[40] Fuda NJ, Guertin MJ, Sharma S, Danko CG, Martins AL, Siepel A, Lis JT (2015). GAGA factor maintains nucleosome-free regions and has a role in RNA polymerase II recruitment to promoters. PLoS Genet.

[41] Vazquez M, Moore L, Kennison JA (1999). The trithorax group gene osa encodes an ARID-domain protein that genetically interacts with the brahma chromatin-remodeling factor to regulate transcription. Development.

[42] Brown JL, Fritsch C, Mueller J, Kassis JA (2003). The Drosophila pho-like gene encodes a YY1-related DNA binding protein that is redundant with pleiohomeotic in homeotic gene silencing. Development.

[43] Lis J (1998). Promoter-associated pausing in promoter architecture and postinitiation transcriptional regulation. Cold Spring Harb Symp Quant Biol.

[44] Zhao K, Hart CM, Laemmli UK (1995). Visualization of chromosomal domains with boundary element-associated factor BEAF-32. Cell.

[45] Gerasimova TI, Corces VG (1996). Boundary and insulator elements in chromosomes. Curr Opin Genet Dev.

[46] Gilmour DS, Lis JT (1986). RNA polymerase II interacts with the promoter region of the noninduced hsp70 gene in Drosophila melanogaster cells. Mol Cell Biol.

[47] Brookes E, Pombo A (2009). Modifications of RNA polymerase II are pivotal in regulating gene expression states. EMBO Rep.

[48] Krämer A, Haars R, Kabisch R, Will H, Bautz FA, Bautz EKF (1980). Monoclonal antibody directed against RNA polymerase II of Drosophila melanogaster. Mol Gen Genet.

[49] Zhang X, Robertson G, Krzywinski M, Ning K, Droit A, Jones S, Gottardo R (2011). PICS: probabilistic inference for ChIP-seq. Biometrics.

[50] Machanick P, Bailey TL (2011). MEME-ChIP: motif analysis of large DNA datasets. Bioinformatics.

[51] van Steensel B, Delrow J, Bussemaker HJ (2003). Genomewide analysis of Drosophila GAGA factor target genes reveals context-dependent DNA binding. Proc Natl Acad Sci USA.

[52] Negre N, Brown CD, Ma L, Bristow CA, Miller SW, Wagner U, Kheradpour P, Eaton ML, Loriaux P, Sealfon R (2011). A cis-regulatory map of the Drosophila genome. Nature.

[53] Oh H, Slattery M, Ma L, Crofts A, White KP, Mann RS, Irvine KD (2013). Genome-wide association of yorkie with chromatin and chromatin-remodeling complexes. Cell Rep.

[54] Huang DW, Sherman BT, Lempicki RA (2008). Systematic and integrative analysis of large gene lists using DAVID bioinformatics resources. Nat Protocols.

[55] Huang DW, Sherman BT, Lempicki RA (2009). Bioinformatics enrichment tools: paths toward the comprehensive functional analysis of large gene lists. Nucleic Acids Res.

[56] Blanch M, Piñeyro D, Bernués J (2015). New insights for Drosophila GAGA factor in larvae. R Soc Open Sci.

[57] Adelman K, Marr MT, Werner J, Saunders A, Ni Z, Andrulis ED, Lis JT (2005). Efficient release from promoter-proximal stall sites requires transcript cleavage factor TFIIS. Mol Cell.

[58] Muse GW, Gilchrist DA, Nechaev S, Shah R, Parker JS, Grissom SF, Zeitlinger J, Adelman K (2007). RNA polymerase is poised for activation across the genome. Nat Genet.

[59] Gaertner B, Johnston J, Chen K, Wallaschek N, Paulson A, Garruss AS, Gaudenz K, De Kumar B, Krumlauf R, Zeitlinger J (2012). Poised RNA polymerase II changes over developmental time and prepares genes for future expression. Cell Reports.

[60] Mavrich TN, Ioshikhes IP, Venters BJ, Jiang C, Tomsho LP, Qi J, Schuster SC, Albert I, Pugh BF (2008). A barrier nucleosome model for statistical positioning of nucleosomes throughout the yeast genome. Genome Res.

[61] Jiang C, Pugh BF (2009). A compiled and systematic reference map of nucleosome positions across the Saccharomyces cerevisiae genome. Genome Biol.

[62] Kaplan N, Moore IK, Fondufe-Mittendorf Y, Gossett AJ, Tillo D, Field Y, LeProust EM, Hughes TR, Lieb JD, Widom J, Segal E (2009). The DNA-encoded nucleosome organization of a eukaryotic genome. Nature.

[63] Ohler U, Liao G-C, Niemann H, Rubin G (2002). Computational analysis of core promoters in the Drosophila genome. Genome Biol.

[64] Zabidi MA, Arnold CD, Schernhuber K, Pagani M, Rath M, Frank O, Stark A (2015). Enhancer-core-promoter specificity separates developmental and housekeeping gene regulation. Nature.

[65] Engström PG, Ho Sui SJ, Drivenes Ø, Becker TS, Lenhard B (2007). Genomic regulatory blocks underlie extensive microsynteny conservation in insects. Genome Res.

[66] Spierer A, Begeot F, Spierer P, Delattre M (2008). SU(VAR)3-7 links heterochromatin and dosage compensation in Drosophila. PLoS Genet.

[67] Adryan B, Teichmann SA (2006). FlyTF: a systematic review of site-specific transcription factors in the fruit fly Drosophila melanogaster. Bioinformatics.

[68] Li J, Liu Y, Rhee HS, Ghosh SK, Bai L, Pugh BF, Gilmour DS (2013). Kinetic competition between elongation rate and binding of NELF controls promoter-proximal pausing. Mol Cell.

[69] Lee H, Kraus KW, Wolfner MF, Lis JT (1992). DNA sequence requirements for generating paused polymerase at the start of hsp70. Genes Dev.

[70] Kwon SY, Xiao H, Glover BP, Tjian R, Wu C, Badenhorst P (2008). The nucleosome remodeling factor (NURF) regulates genes involved in Drosophila innate immunity. Dev Biol.

[71] Pointud J-C, Larsson J, Dastugue B, Couderc J-L (2001). The BTB/POZ domain of the regulatory proteins Bric à brac 1 (BAB1) and Bric à brac 2 (BAB2) Interacts with the Novel Drosophila TAFII factor BIP2/dTAFII155. Dev Biol.

[72] Chopra VS, Srinivasan A, Kumar RP, Mishra K, Basquin D, Docquier M, Seum C, Pauli D, Mishra RK (2008). Transcriptional activation by GAGA factor is through its direct interaction with dmTAF3. Dev Biol.

[73] Chang Y-L, King B, Lin S-C, Kennison JA, Huang D-H (2007). A double-bromodomain protein, FSH-S, activates the homeotic gene ultrabithorax through a critical promoter-proximal region. Mol Cell Biol.

[74] Johansen KM, Cai W, Deng H, Bao X, Zhang W, Girton J, Johansen J (2009). Polytene chromosome squash methods for studying transcription and epigenetic chromatin modification in Drosophila using antibodies. Methods.

[75] Huang D-H, Chang Y-L, Yang C-C, Pan IC, King B (2002). pipsqueak encodes a factor essential for sequence-specific targeting of a polycomb group protein complex. Mol Cell Biol.

[76] Fuda NJ, Buckley MS, Wei W, Core LJ, Waters CT, Reinberg D, Lis JT (2012). Fcp1 dephosphorylation of the RNA polymerase II C-terminal domain is required for efficient transcription of heat shock genes. Mol Cell Biol.

[77] Gilchrist DA, Fargo DC, Adelman K (2009). Using ChIP-chip and ChIP-seq to study the regulation of gene expression: genome-wide localization studies reveal widespread regulation of transcription elongation. Methods.

[78] Petesch SJ, Lis JT (2008). Rapid, transcription-independent loss of nucleosomes over a large chromatin domain at Hsp70 Loci. Cell.

