# Supplementary material for: GAGA factor, a positive regulator of global gene expression, modulates transcriptional pausing and organization of upstream nucleosomes
Source: Epigenetics Chromatin. 2016 Jul 27;9:32. doi: 10.1186/s13072-016-0082-4 (PMC4962548; doi:10.1186/s13072-016-0082-4)
Supplement: Supplementary file 1 — 10.1186/s13072-016-0082-4 Figure S1. Analyses of RNA-Pol in Gaf mutants. Figure S2. RNA-Pol Hypo-p and Ser-2p are not affected by GAF on polytene chromosomes. Figure S3. Comparison of GAF target genes identified in different reports. Figure S4. Verification of gene expression by microarray studies. Figure S5. Procedures of statistical analyses of GAF and RNA-Pol ChIP-seq data. Figure S6. The abundance of different GAF binding motifs among targets. Figure S7. Reduction promoterproximal RNA-Pol in the Gaf mutant. Figure S8. Comparison of paused genes identified in different reports. Figure S9. Comparion of nucleosome profiles. Figure S10. Analyses of GAF’s effects on eye development. Figure S11. Comparison of target genes for GAF, NELF and NURF. [file 13072_2016_82_MOESM1_ESM.pdf]

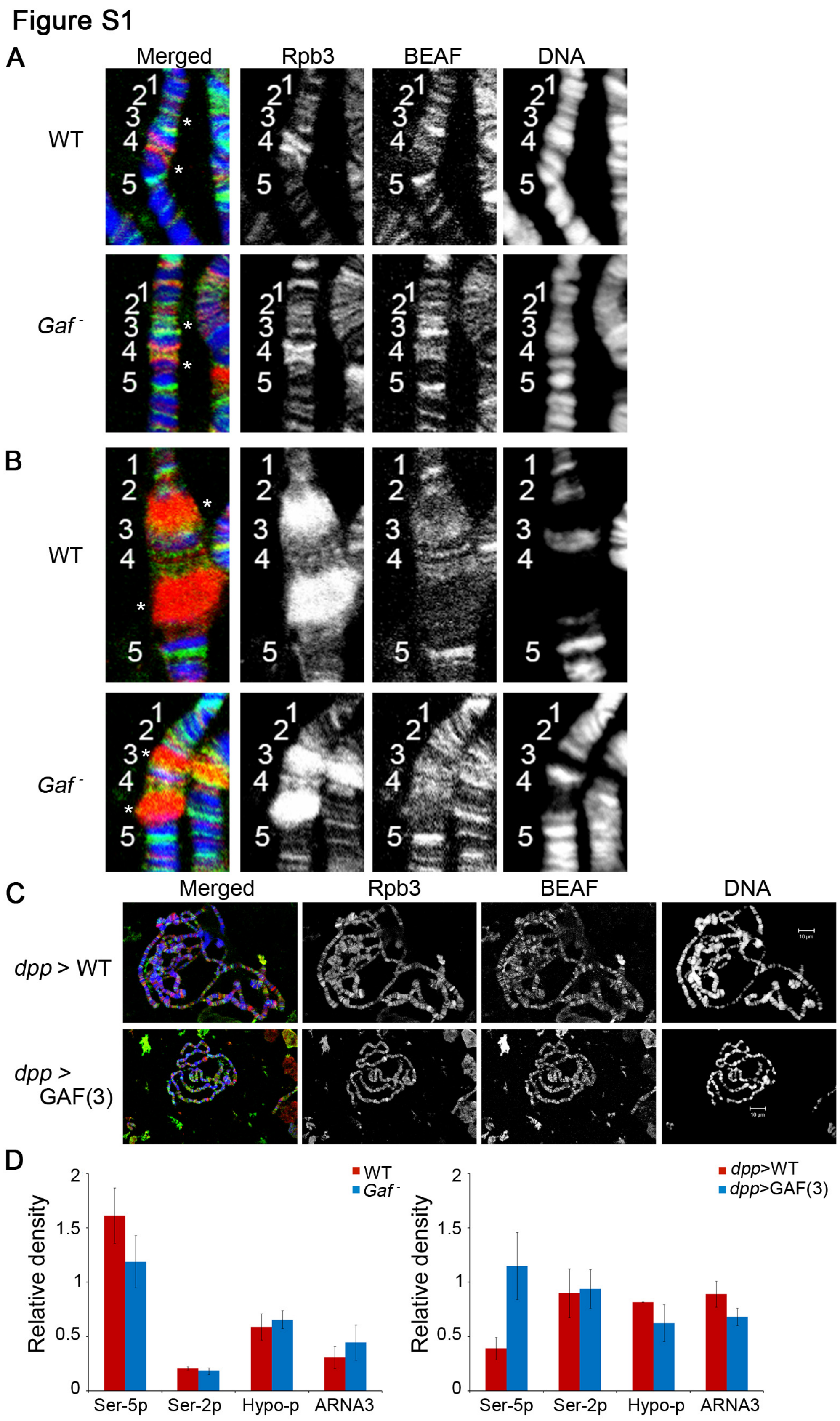

Figure S1. Analyses of RNA-Pol in *Gaf* mutants.

A-C. Cytogenetic studies of total RNA-Pol at *Hsp70* loci. Polytene chromosomes prepared from the following samples were co-stained with antibodies against Rpb3 (red) and BEAF-32 (green): WT and mutants before (A), after 5-min heat shock (B), and *dpp* > WT or *dpp* > GAF(3) (C). The markings of the region from 87A to 87C is described in Fig. 1D-F.

D. Quantitation of different RNA-Pol isoforms. Immunoblots shown in Fig 2C were quantified. The relative abundance of each RNA-Pol isoform was adjusted with that of α-tubulin. The bar graphs show the averages of triplicate experiments.

Figure S2

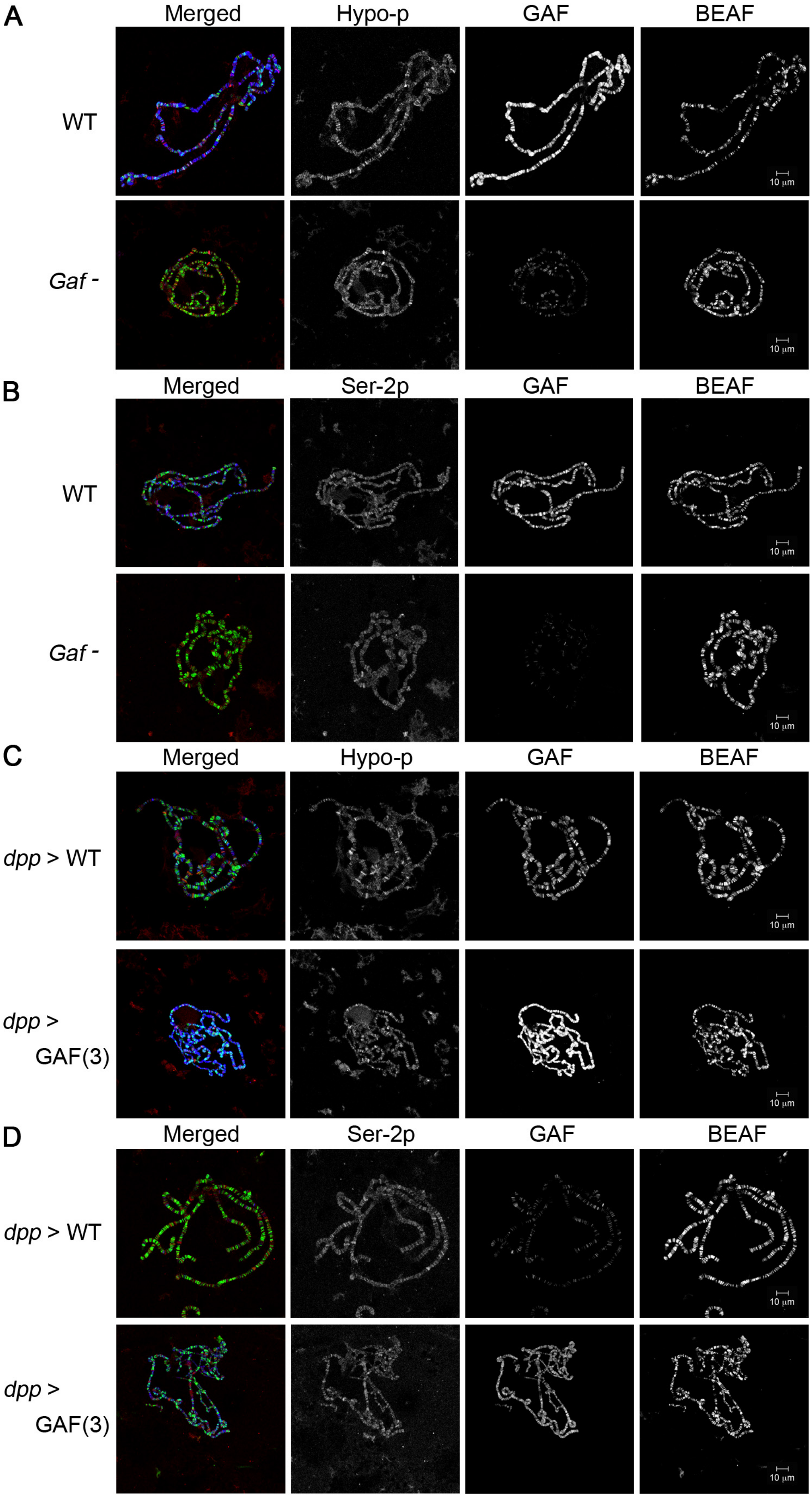
A
Merged
Hypo-p
GAF
BEAF
WT
Gaf -
B
Merged
Ser-2p
GAF
BEAF
WT
Gaf -
C
Merged
Hypo-p
GAF
BEAF
dpp > WT
dpp > GAF(3)
D
Merged
Ser-2p
GAF
BEAF
dpp > WT
dpp > GAF(3)
10 µm

Figure S2. RNA-Pol Hypo-p and Ser-2p are not affected by GAF on polytene chromosomes.

Polytene chromosomes from the WT and *Gaf* mutant (A, B) or from *dpp* > WT and *dpp* > GAF(3) lines (C, D) were co-stained with RNA-Pol Hypo-p (A, C) or Ser-2p antibodies (B, D) (in red), along with GAF (in blue), and BEAF-32 (in green) antibodies. The colors are adjusted to the grey scale for individual panels as in Fig. 1D-F.

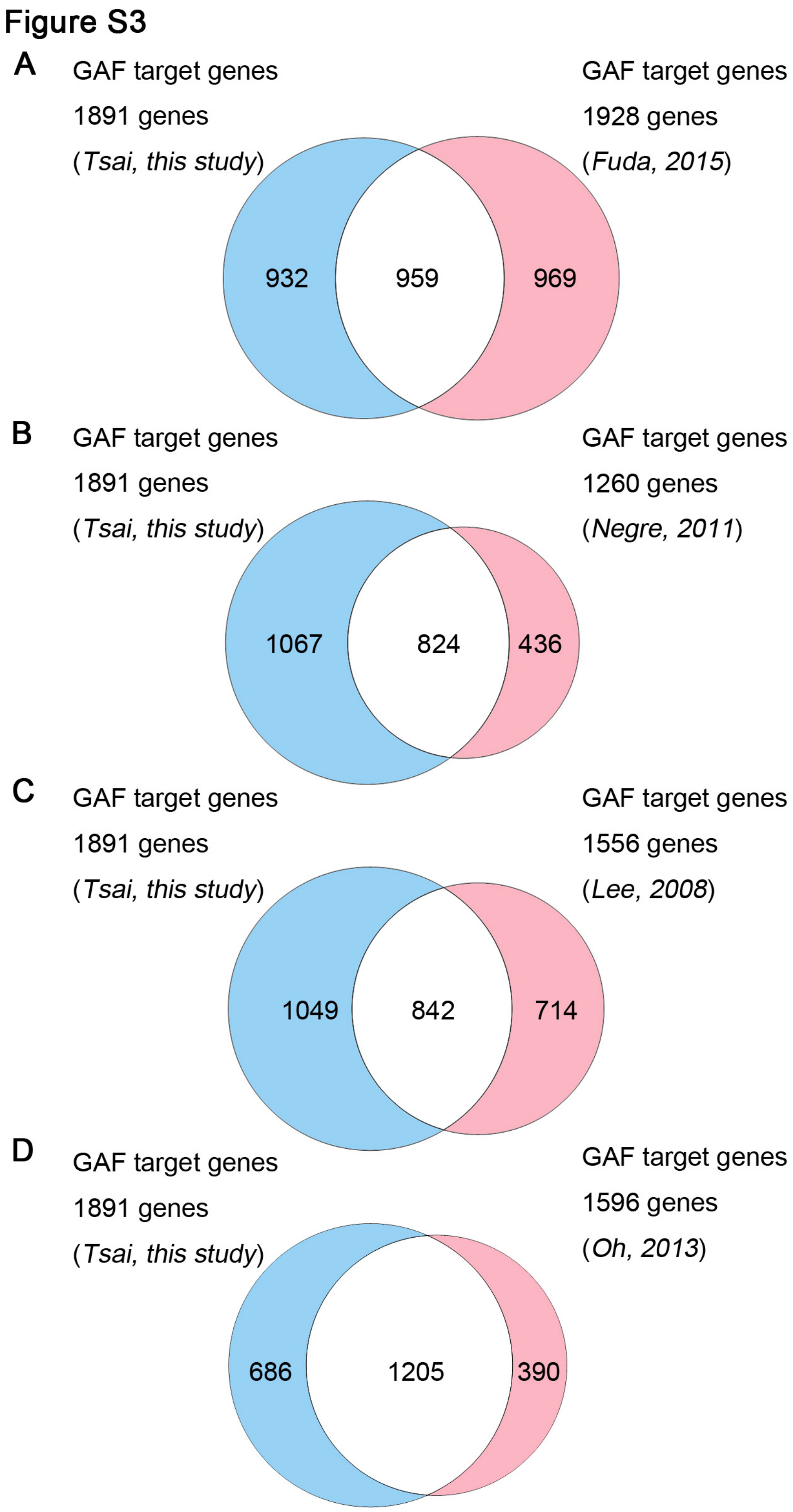

Figure S3. Comparison of GAF target genes identified in different reports.

The collection of each dataset is represented as a Venn diagram. GAF target genes identified in our study (blue) are compared with the collection identified earlier

(pink) by Fuda *et al.*, 2015 (A), Negre *et al.*, 2011 (B, dataset for 16-24 hr embryo), Lee *et al.*, 2008 (C), or Oh *et al.*, 2013 (D). Common targets are shown in white.

Figure S4

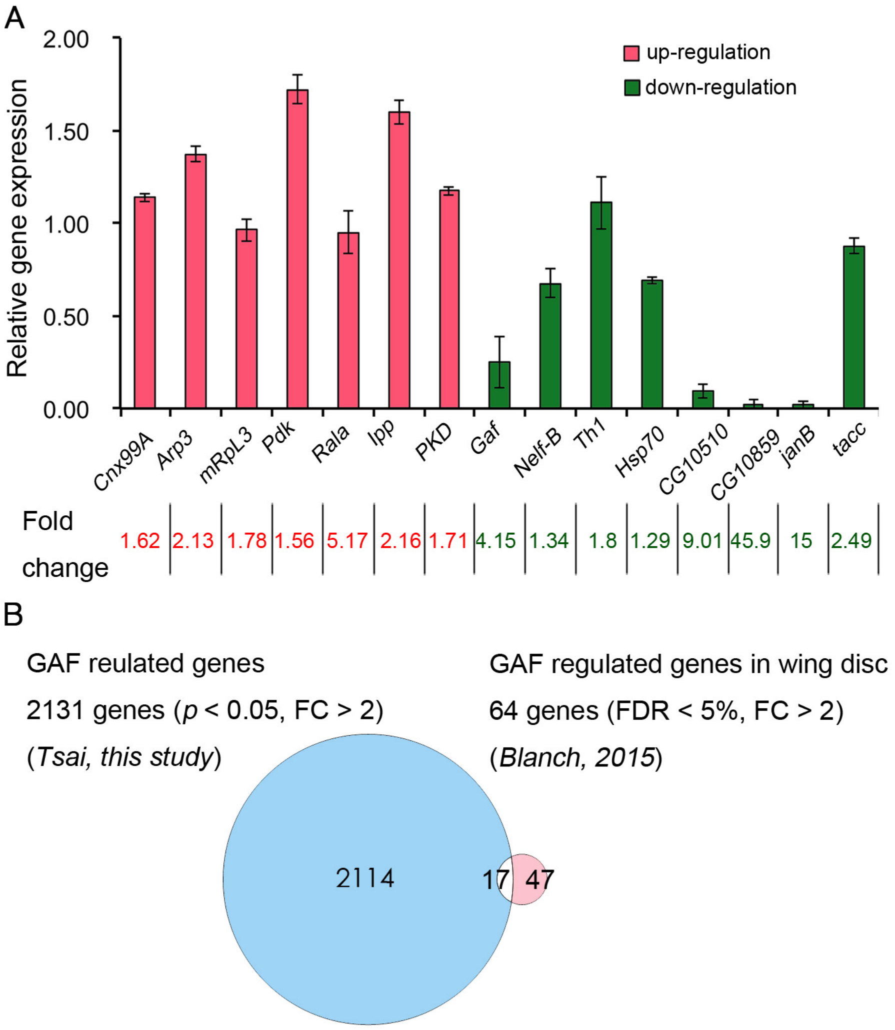

Figure S4. Verification of gene expression by microarray studies.

A. RT-qPCR was performed to verify the change in RNA level observed in microarray experiments. RNAs from WT and mutant samples were reverse-transcribed, and the amounts of cDNAs were determined by qPCR. The expression level of 7 up-regulated (red bar) and 8 down-regulated genes (green bar) was calibrated with that of actin. The relative gene expression represents the ratio between mutant and WT samples for each gene. The fold change observed in microarray studies is shown below. The average of triplicate qPCR experiments is shown.

B. Comparsion of gene expression data. GAF-regulated genes identified in our study (blue) are compared with the collection identified earlier (pink) by Blanch *et al.*, 2015. Common targets are shown in white.

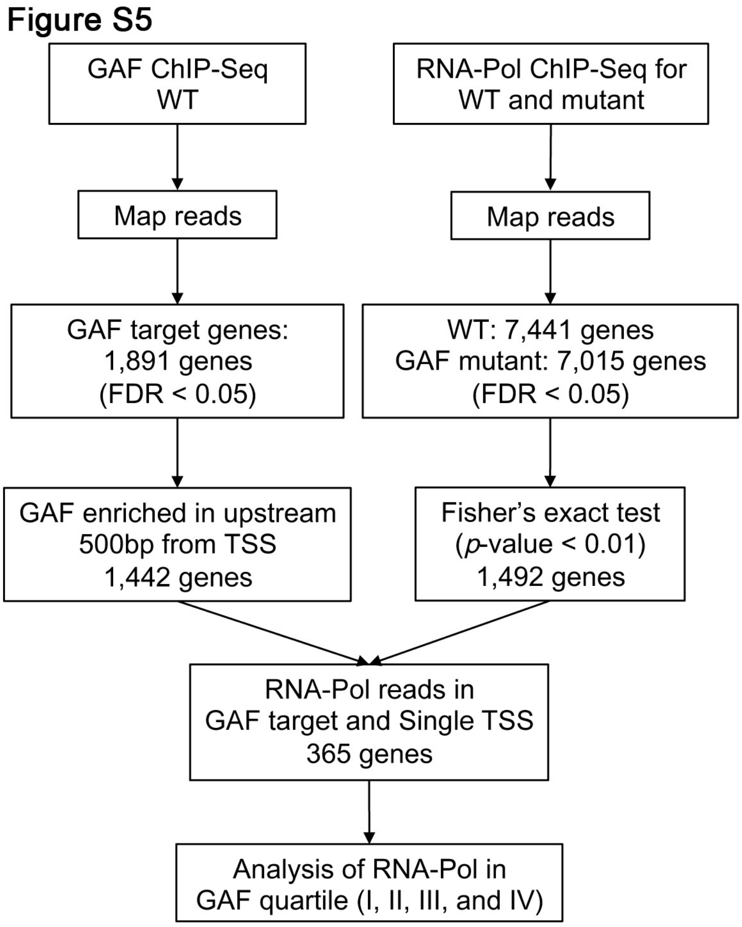

Figure S5. Procedures of statistical analyses of GAF and RNA-Pol ChIP-seq data.

Raw reads from ChIP-seq were mapped to the fly genome and then selected according to their statistical values (FDR < 0.05), yielding 1891 genes with GAF peaks and ~7000 genes (7441 and 7015 genes in the WT and mutant, respectively) with RNA-Pol. Further selection was conducted to identify those with enriched GAF peaks (> 2-fold over input) in the 500 bp upstream region, yielding 1442 GAF target genes. Independently, 1492 genes with significant amounts of RNA-Pol in the transcribed region ($p < 0.01$ in Fisher's exact test) were identified, followed by further selection by three criteria: (1) single TSS, (2) no other gene within 1 kb of TSS, (3) transcribed region larger than 0.8 kb. 365 genes fulfilling both criteria for GAF and RNA-Pol were then subjected to analyses for RNA-Pol density or PI as a whole or in four separate ranks.

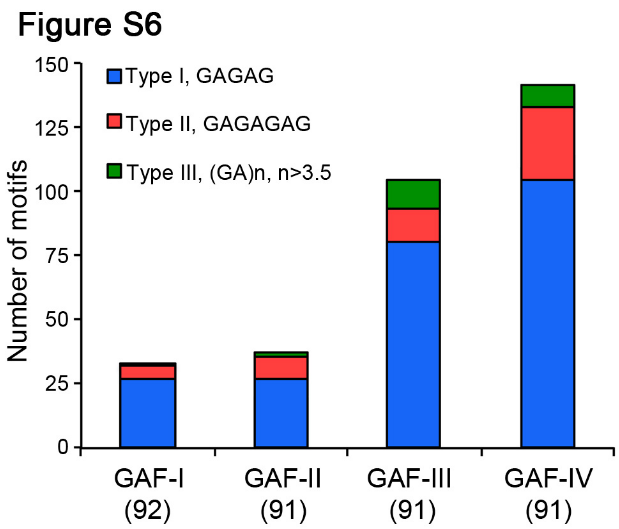

Figure S6. The abundance of different GAF binding motifs among targets.

GAF binding motifs are simplified into three major types: Type I, GAGAG (blue); Type II, GAGAGAG (red); Type III, (GA)n, n>3.5 (green). The abundance of these motifs is represented by stacked columns. The number of genes in each rank is indicated.

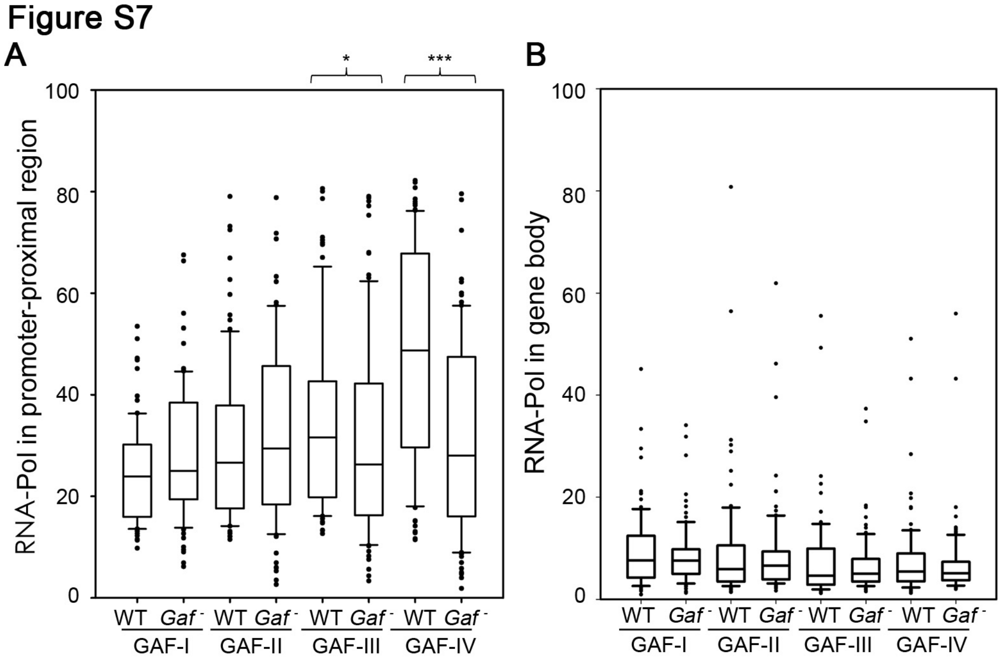

Figure S7. Reduction promoter-proximal RNA-Pol in the *Gaf* mutant.

RNA-Pol density in the promoter-proximal region (A), or gene body (B) is presented in box plots for each rank of genes in WT and mutants. The whiskers indicate 5% and 95% level of RNA-Pol density for each plot. The outliers are also shown. Statistical significance of differences calculated by Mann-Whitney U test is indicated for ranks III (p = 0.035, *) and IV (p = 3.93x10^{-7}, ***) for the promoter-proximal region.

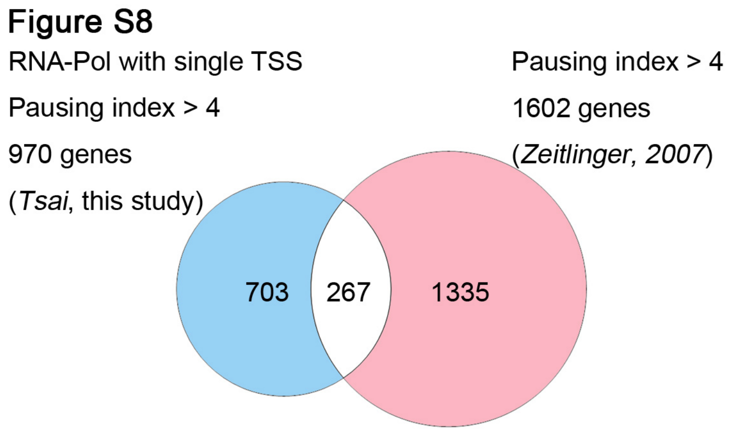

Figure S8. Comparison of paused genes identified in different reports.

Genes with a pausing index higher than 4 were selected from our collection (blue) and from Zeitliger *et al.*, 2007 (pink). Paused genes represented in the Venn diagram are compared. Common targets are shown in white.

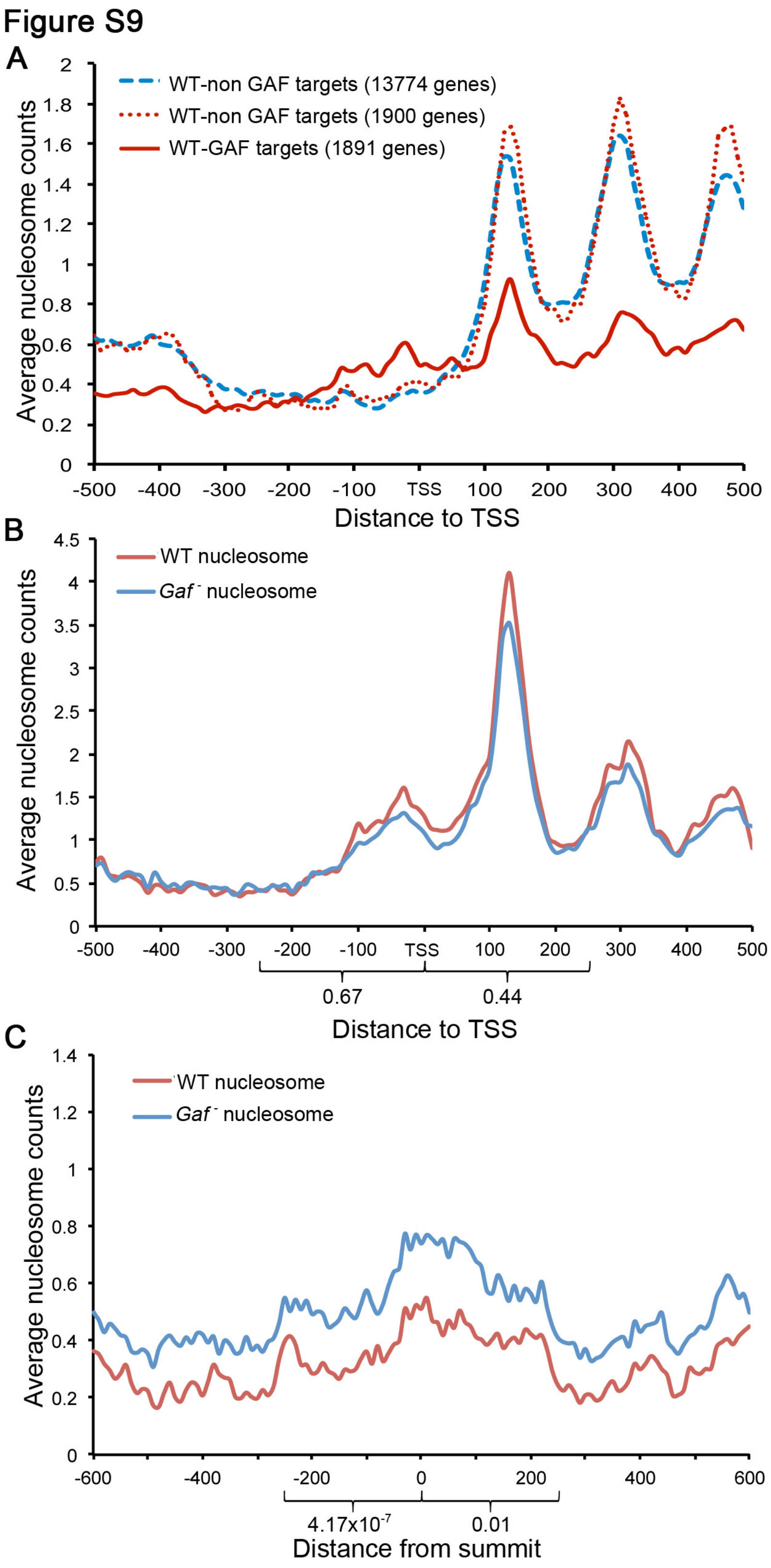

Figure S9. Comparion of nucleosome profiles.

A. The overall nucleosome distribution of 13774 (blue dash line) or 1900 (red dash line) non-targets in WT genome is shown for the 1 kb region around

TSS. The nucleosome distribution of 1891 GAF targets (red solid line) is also included for the reference. The 1900 non-targets were collected by randomly choosing 380 genes from each of five chromosomal arms.

B. Nucleosome profile and RNA-Pol in non-targets. 210 non-target genes with > 20% reduction of promoter-proximal RNA-Pol in *Gaf* mutant were selected. The nucleosome profiles of these genes in WT and *Gaf* mutant are shown. No significant difference is seen. The average nucleosome counts were calculated by the same method described in Fig. 4A. The statistical differences between these two patterns were calculated by Kolmogorov-Smirnov tests.

C. Nucleosome profiles around intergenic GAF peaks. 516 intergenic GAF peaks were selected for analyses. The nucleosome profiles around GAF peaks are shown for WT and *Gaf* mutant. Kolmogorov-Smirnov tests was performed.

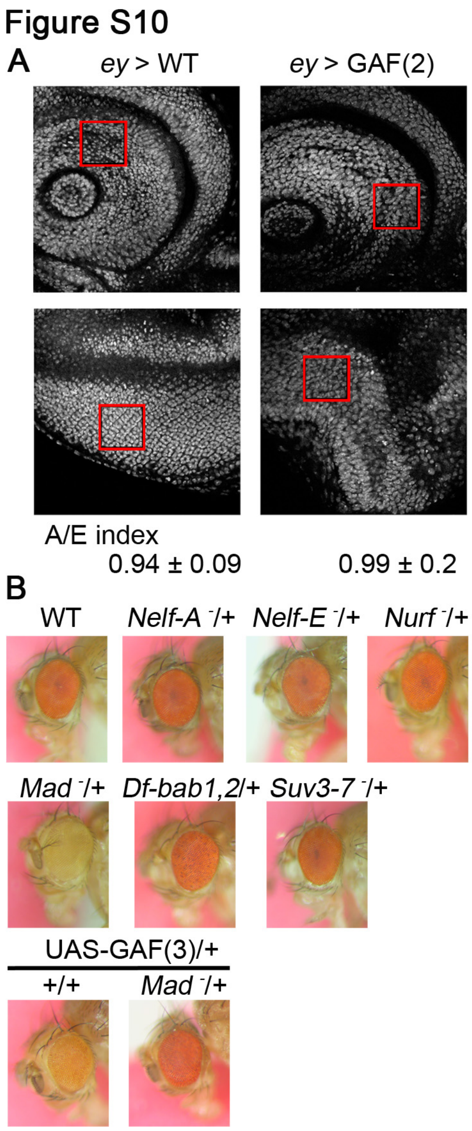

Figure S10. Analyses of GAF's effects on eye development.

A. Comparison of eye-antenna discs from *ey* > WT and *ey* > GAF(2) larvae. The eye-antenna imaginal discs were fixed and stained for DNA. To obtain the relative nuclei density between antenna and posterior eye discs (A/E index), nuclei numbers were measured based on DNA staining in antenna discs (upper panel) or posterior eye (lower panel) discs for the same space (red box, 9×10^2 μm^2). The ratio of nuclei density between antenna (A) and eye (E) discs in *ey* > WT and *ey* > GAF(2) larvae was calculated. The average ratios from 15 discs are given.

B. Genetic test for GAF-interacting factors. The upper and middle panels show the typical adult eyes in WT and mutants. The lower panel shows adult eyes from UAS-GAF(3) on the third chromosome.

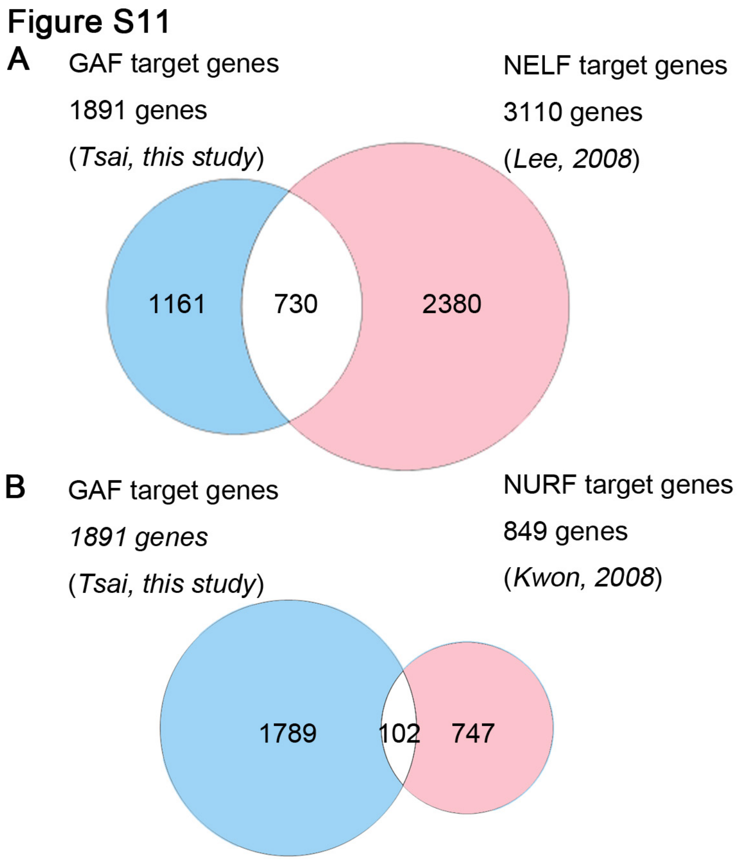

Figure S11. Comparison of target genes for GAF, NELF and NURF.

GAF target genes identified in our study (blue) are compared with NELF targets identified by Lee *et al.*, 2008 (A, pink), or NURF targets identified by Kwon *et al.*, 2008 (B, pink) in Venn diagrams. Common targets are shown in white.
