# Supplementary material for: GAGA factor, a positive regulator of global gene expression, modulates transcriptional pausing and organization of upstream nucleosomes
Source: Epigenetics Chromatin. 2016 Jul 27;9:32. doi: 10.1186/s13072-016-0082-4 (PMC4962548; doi:10.1186/s13072-016-0082-4)
Supplement: Supplementary file 2 — 10.1186/s13072-016-0082-4 Table S1. Putative transcription factors identified in GAF target genes. Table S2. PCR primers used for ChIP analysis of Hsp70. Table S3. Primers used for RT-qPCR. [file 13072_2016_82_MOESM2_ESM.pdf]

Supplemental Tables

Table S1.

Putative transcription factors identified in GAF target genes.

FlyTF database was used to screen GAF target genes (FDR < 0.05, enrichment > 2) with more than 1.5-fold change in gene expression.

	FBgn	GAF enrichment	Symbol
1	FBgn0004652		fru
2	FBgn0002723		Met
3	FBgn0004394		pdm2
4	FBgn0085432		pan
5	FBgn0004870		bab1
6	FBgn0000448		Hr46
7	FBgn0261238		Alh
8	FBgn0039938		Sox102F
9	FBgn0040318		HGTX
10	FBgn0005630	GAF > 2	lola
11	FBgn0034240		MESR4
12	FBgn0010228		HmgZ
13	FBgn0011656		Mef2
14	FBgn0014179		gcm
15	FBgn0011758		B-H1
16	FBgn0001147		gsb-n
17	FBgn0015721		king-tubby
18	FBgn0031894		CG4496
19	FBgn0041111		lilli
20	FBgn0004606		zfh1

21	FBgn0085369		CG34340
22	FBgn0020617		Rx
23	FBgn0015396		jumu
24	FBgn0085451		CG34422
25	FBgn0050403		CG30403
26	FBgn0026160		tna
27	FBgn0002576		lz
28	FBgn0000577		en
29	FBgn0011764		Dsp1
30	FBgn0000504		dsx
31	FBgn0004362		HmgD
32	FBgn0008651		lbl
33	FBgn0036423		CG3919
34	FBgn0261239		Hr39
35	FBgn0016694		Pdp1
36	FBgn0004858		elB
37	FBgn0028979		tio
38	FBgn0259211		grh
39	FBgn0042650		disco-r
40	FBgn0259938		cwo

Table S2.

PCR primers used for ChIP analysis of *Hsp70*.

Primer name	5’ to 3’
−182 forward	GGCAGAAAGAAAACTCGAGAAATTT
−182 reverse	AAGAGGGAGAGTCACAAAACGAAT
+58 forward	CAATTCAAACAAGCAAAGTGAACAC
+58 reverse	TGATTCACTTTAACTTGCACTTTA
+379 forward	CACCACGCCGTCCTACGT
+379 reverse	GGTTCATGGCCACCTGGTT
+1702 forward	GGGTGTGCCCCAGATAGAAG
+1702 reverse	TGTCGTTCTTGATCGTGATGTTC

Table S3.

Primers used for RT-qPCR.

A list of PCR primers used for quantitative measurement of the RNA level.

Primer name	5’ to 3’
Actin42A forward	CGCTCATTTCCGATGGTGAT
Actin42A reverse	TGCTACGTGGCCTTGGACTT
Cnx99A forward	TTCAAGGACAAGCTACCCCATCT
Cnx99A reverse	GATGCGATGCAGCTGAGATC
Arp3 forward	CGCCAAAGAATTTGCCAAGT
Arp3 reverse	GCCGCTAAAGTTGCGTATCC
mRpL3 forward	CCACCGTTCCCAACGTTTT
mRpL3 reverse	CACCTGTTCGTACCACACATCAT
Pdk forward	AGGGTGTGATCGAAATGAAGGA
Pdk reverse	AACTCTCTGTAGGCGCATCCA
Rala forward	TAAGATCACGAAAAACCGAAGATTC
Rala reverse	CTTAAGTCTCCGCTTCTTGCATCTAT
Ipp forward	GCTCATCCAGGAGACGATCAA
Ipp reverse	CCAGGATGGCATCCTTCATG
PKD forward	TCGTCAATCTGGAGCGAATG
PKD reverse	TTGAGCTTTTCCATCACCACAA
GAF forward	CTGCTATCATCCACACCGATCA
GAF reverse	GGGTCCATGGTCACAATAGTCTCT
Nelf-B forward	CTGCACCTCGAGATCGTGTAAG
Nelf-B reverse	GCGCACCCGCAATGTT
TH1 forward	CCTGGACTTCGGTAACCTTGAC
TH1 reverse	CCAGATGGCCAACCTGCTT
Hsp70 forward	GGGTGTGCCCCAGATAGAAG
Hsp70 reverse	TGTCGTTCTTGATCGTGATGTTC
CG10510 forward	CGCCCCAAGCCCTCAT

CG10510 reverse	TGTGCCGCGAAATCAAGA
CG10859 forward	CATAATGTTCACCTCCTTGACGTT
CG10859 reverse	GATTCCCATGGCTTGAATCACT
janB forward	CGTGAAGCCATGCGTATGAATA
janB reverse	CCCCGGGTCAAGATAACCA
tacc forward	AGATGACCTCGCAGCTTAAAAGTAAC
tacc reverse	GATTGTCCATCATCTGCTTTCG
