# Supplementary material for: GAGA factor, a positive regulator of global gene expression, modulates transcriptional pausing and organization of upstream nucleosomes
Source: Epigenetics Chromatin. 2016 Jul 27;9:32. doi: 10.1186/s13072-016-0082-4 (PMC4962548; doi:10.1186/s13072-016-0082-4)
Supplement: Supplementary file 3 — 10.1186/s13072-016-0082-4 Supplementary Methods. [file 13072_2016_82_MOESM3_ESM.pdf]

Supplemental methods

ChIP-seq alignment

Raw sequences were aligned to the genome sequence in *Drosophila* Release 5.47 using CLC-bio (Qiagen, http://www.clcbio.com/). Peaks of ChIP-enriched sequences were identified using PICS (Probabilistic Inference for ChIP-Seq, Avadis-NGS, http://www.avadis-ngs.com) with a false discovery rate (FDR) set at < 0.05 [1]. For GAF ChIP-seq, 29630021 and 30460578 unique reads were mapped for input and ChIP samples, respectively. For RNA-Pol ChIP-seq, 31672824, 27343320 and 28160920 unique reads were mapped for input, WT, and *Gaf* mutant samples, respectively. After adjustment for different data sizes, ChIP-seq reads were further normalized using RPKM (reads per kilobase per million) for each data set [2].

Calculation of RNA-Pol density and pausing index

To avoid repetitive computation for RNA-Pol reads in GAF target genes, we screened among 7939 genes using the following criteria: (1) single TSS; (2) no overlapping gene within 1 kb region of TSS; (3) gene size larger than 800 bp. Fisher's exact test was used to evaluate the significance of RNA-Pol reads for each gene by comparing RNA-Pol density in the promoter-proximal region (from -100 to +150) with that in the nearby intergenic region for both input and RNA-Pol ChIP. Based on the score of Fisher's test ($p < 0.01$), 1492 genes were selected for further comparison. The PI was calculated according to a published procedure [3]. The RNA-Pol density of the promoter-proximal region and gene body region (from +500 to the gene end) was calculated and used as numerator and denominator, respectively.

Mapping of promoter-proximal nucleosomes

After mapping of unique reads obtained from paired-end sequencing, data corresponding to fragment sizes between 120~180 bp were collected, yielding total reads of 47980833 and 57867511 for the WT and *Gaf* mutant, respectively. The center of each read was designated as the position of the nucleosome. Due to the large variation in nucleosome distribution around TSS and the immediate upstream region, we used the following formula to normalize total reads of WT and *Gaf* mutant samples within the region from -500 to +500 and calculate average nucleosome counts for each nucleotide coordinate.

$$\frac{A'}{\left(\frac{A}{A+B}\right)} \text{ and } \frac{B'}{\left(\frac{B}{A+B}\right)}$$

A: total reads of WT sample.

A’: cumulative reads at each nucleotide coordinate for WT sample.

B: total reads of mutant sample.

B’: cumulative reads at each nucleotide coordinate for mutant sample.

The sample under normalization was used as the numerator.

The average nucleosome count was plotted against the nucleotide coordinate from -500 to +500 of the metagene. Data are presented as 10-bp intervals.

Gene ontology analysis

Database for Annotation, Visualization and Integrated Discovery (DAVID) was employed [4, 5]. Functional annotation clustering was set at high stringency to deduce Biological Processes (BP) of GAF target genes.

Motif analysis of GAF targets

DNA sequences of GAF peaks from ChIP-seq experiments were searched on both strands for enriched motifs using MEME-ChIP [6]. The length of the motif was set between 5 bp and 30 bp.

Predicted nucleosome propensity

The prediction of nucleosome propensity was based on the presence of consecutive SS-dinucleotides (GC, GG, CG, CC), according to previous study on the genome-wide nucleosome distribution of *Drosophila* [7]. A simplified method was adopted to calculate SS-dinucleotides content of DNA sequences from -500 to +500. Any of the four combinations of SS-dinucleotides was given a value of one. Cumulative values for each dinucleotide unit were normalized to the gene number and plotted against the nucleotide coordinate to generate the predicted nucleosome distribution of the metagene for each group. Heat maps were generated with R project [8].

Analysis of motif enrichment for core promoters

We used MEME Suite to screen for motifs of core promoters and transcription factors in datasets established for GAF target and non-target genes from our ChIP analyses [6]. Both strands of DNA sequences from ±300 around TSS of these genes were analyzed by MCAST for a match ($p < 0.005$) to TATA box, Inr, MTE, DPE, and Ohler motifs 1, 5, 6, 7 [9, 10].

Identification of putative transcription factors

A search for transcription factor (TF) binding motifs was performed using MEME-ChIP for different groups of GAF target genes separately [6]. Both strands of

DNA sequences from -1,000 to +500 of these genes were analyzed with the limit of motif length set at 20 bp. Conserved motifs were subsequently used to identify putative transcription factors ($q < 0.01$) using TOMTOM. The putative TFs were further verified with the FlyTF database containing 753 validated TFs [11].

Histology of the adult eyes

Adult heads were collected and soaked in 100% isopropanol for two days with several changes at room temperature. After dehydration, heads were stuck onto stubs and coated by Cressington Sputter Coater 108 (Ted Pella, Inc.). Samples were viewed and photographed with an FEI Quanta 200 scanning electron microscope (FEI, Eindhoven) at the ICOB Imaging Core Facility.

Imaginal disc staining

Eye-antenna discs were isolated from third instar larvae raised at 21°C before dissection. Discs were fixed and DNA was stained with Hoechst 33258 (0.25mg/ml) (Polysciences, 09460). A Zeiss LSM 510 confocal microscope was used to detect the fluorescent staining.

Supplemental references

1. Zhang X, Robertson G, Krzywinski M, Ning K, Droit A, Jones S, Gottardo R: **PICS: Probabilistic Inference for ChIP-seq.** *Biometrics* 2011, **67:**151-163.
2. Wagner G, Kin K, Lynch V: **Measurement of mRNA abundance using RNA-seq data: RPKM measure is inconsistent among samples.** *Theory in Biosciences* 2012, **131:**281-285.
3. Nechaev S, Fargo DC, dos Santos G, Liu L, Gao Y, Adelman K: **Global Analysis of Short RNAs Reveals Widespread Promoter-Proximal Stalling and Arrest of Pol II in Drosophila.** *Science* 2010, **327:**335-338.
4. Huang DW, Sherman BT, Lempicki RA: **Systematic and integrative analysis of large gene lists using DAVID bioinformatics resources.** *Nat Protocols* 2008, **4:**44-57.
5. Huang DW, Sherman BT, Lempicki RA: **Bioinformatics enrichment tools: paths toward the comprehensive functional analysis of large gene lists.** *Nucleic Acids Research* 2009, **37:**1-13.
6. Machanick P, Bailey TL: **MEME-ChIP: motif analysis of large DNA datasets.** *Bioinformatics* 2011, **27:**1696-1697.
7. Mavrich TN, Jiang C, Ioshikhes IP, Li X, Venters BJ, Zanton SJ, Tomsho LP, Qi J, Glaser RL, Schuster SC, et al: **Nucleosome organization in the Drosophila genome.** *Nature* 2008, **453:**358-362.
8. Team RC: **R: A language and environment for statistical computing.** *R Fiundation for Statistical Computing, Vienna, Austria* 2014.
9. Ohler U, Liao G-c, Niemann H, Rubin G: **Computational analysis of core promoters in the Drosophila genome.** *Genome Biology* 2002, **3:**1 -12.
10. Zabidi MA, Arnold CD, Schernhuber K, Pagani M, Rath M, Frank O, Stark A:

Enhancer-core-promoter specificity separates developmental and housekeeping gene regulation. *Nature* 2015, **518:**556-559.

11. Adryan B, Teichmann SA: **FlyTF: a systematic review of site-specific transcription factors in the fruit fly Drosophila melanogaster.** *Bioinformatics* 2006, **22:**1532-1533.
